# NEXN regulates vascular smooth muscle cell phenotypic switching and neointimal hyperplasia

**DOI:** 10.1172/jci.insight.190089

**Published:** 2025-05-29

**Authors:** Zexuan Lin, Chaojie Wang, Zhuohua Wen, Zhaohui Cai, Wenjie Guo, Xin Feng, Zengyan Huang, Rongjun Zou, Xiaoping Fan, Canzhao Liu, Hanyan Yang

**Affiliations:** 1 Department of Cardiology, Laboratory of Heart Center, Translational Medicine Research Center, Zhujiang Hospital, Southern Medical University, Guangzhou, China.; 2 Guangdong Provincial Key Laboratory of Cardiac Function and Microcirculation, Guangdong Provincial Biomedical Engineering Technology Research Center for Cardiovascular Disease, Guangzhou, China.; 3 Department of Cardiovascular Surgery, Guangdong Provincial Hospital of Chinese Medicine, The Second Affiliated Hospital of Guangzhou University of Chinese Medicine, Guangzhou, China.; 4 Neurosurgery Center, Department of Cerebrovascular Surgery, Engineering Technology Research Center of Education Ministry of China on Diagnosis and Treatment of Cerebrovascular Disease, Zhujiang Hospital, Southern Medical University, Guangzhou, China.; 5 Department of Pharmacology, Zhongshan School of Medicine, Sun Yat-sen University, Guangzhou, China.; 6 Department of Traditional Chinese Medicine, Zhujiang Hospital, Southern Medical University, Guangzhou, China.

**Keywords:** Cell biology, Vascular biology, Cardiovascular disease, Cell cycle, Cytoskeleton

## Abstract

Vascular smooth muscle cells (VSMCs) exhibit substantial heterogeneity and plasticity, enabling them to switch between contractile and synthetic states, which is crucial for vascular remodeling. Nexilin (NEXN) has been identified as a high-confidence gene associated with dilated cardiomyopathy. Existing evidence indicates NEXN is involved in phenotypic switching of VSMCs. However, a comprehensive understanding of the cell-specific roles and precise mechanisms of NEXN in vascular remodeling remains elusive. Using integrative transcriptomics analysis and smooth muscle–specific lineage-tracing mice, we demonstrated NEXN was highly expressed in VSMCs, and the expression of NEXN was significantly reduced during the phenotypic transformation of VSMCs and intimal hyperplasia induced by vascular injury. VSMC-specific NEXN deficiency promoted the phenotypic transition of VSMCs and exacerbated neointimal hyperplasia in mice following vascular injury. Mechanistically, we found NEXN primarily mediated VSMC proliferation and phenotypic transition through endoplasmic reticulum (ER) stress and Krüppel-like factor 4 signaling. Inhibiting ER stress ameliorated VSMC phenotypic transition by reducing cell cycle activity and proliferation caused by NEXN deficiency. These findings indicate targeting NEXN could be explored as a promising therapeutic approach for proliferative arterial diseases.

## Abstract 2

NEXN deficiency predominantly promotes cell cycle progression and proliferation through the ER stress-KLF4 signal, thereby modulating VSMCs phenotypic transition, and contributing to neointimal hyperplasia.

## Graphical abstract

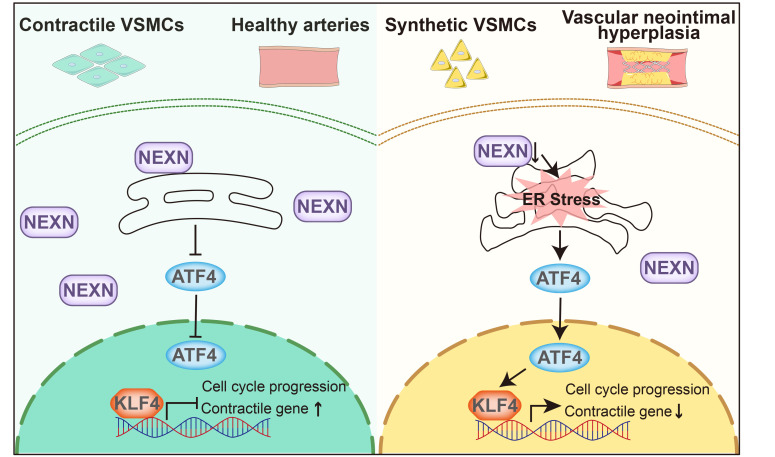

## Introduction

Percutaneous coronary intervention (PCI) and stent placement serve as the preferred methods for treating obstructive arterial disease. However, vascular injury during surgery induces phenotypic transition of vascular smooth muscle cells (VSMCs), leading to serious complications, such as in-stent restenosis and neointimal hyperplasia, which significantly impair patient prognosis (1, 2). VSMCs display remarkable heterogeneity and plasticity, which allow them to adapt their phenotype in response to environmental cues. Their ability to switch from a contractile to a synthetic state is critical for processes like vascular repair, remodeling, and disease pathogenesis. The regulation of VSMC phenotype is highly complex and influenced by a broad range of molecules, including membrane receptors, ion channels, microRNAs, the cytoskeleton, and extracellular matrix (3–5). Despite advances, the intricate molecular mechanisms governing VSMC phenotypic switching remain incompletely understood.

The actin cytoskeleton of VSMCs serves as a dynamic framework essential for generating mechanical tension and maintaining vascular luminal integrity. Following arterial injury, the VSMC cytoskeleton undergoes maladaptive remodeling, triggering multiple signaling pathways that lead to changes in VSMC activities, including proliferation, migration, contraction, and gene expression (6). Thus, elucidating the mechanisms by which the actin cytoskeleton regulates VSMC phenotypic transition is crucial for developing effective therapeutic strategies to improve clinical outcomes following PCI.

Nexilin, encoded by the *NEXN* gene, was initially identified as an actin filament (F-actin) binding protein (7). Mutations in the *NEXN* gene have been linked to dilated and hypertrophic cardiomyopathies in humans (8–10). Our previous studies have shown that NEXN is a component of the junctional membrane complex in cardiomyocytes and plays a critical role in T-tubule initiation and formation. Homozygous deletion of the NEXN G650 residue causes cardiomyopathy in mice, while adeno-associated virus–mediated *NEXN* gene delivery can restore cardiomyocyte function (11–14).

Recent evidence has underscored the significant role of NEXN in maintaining the contractile phenotype of VSMCs. In vitro experiments indicated that NEXN knockdown reduces the expression of SMC contractile markers (15). Notably, genetic findings have identified a variant of NEXN associated with the susceptibility to coronary artery disease (16). These findings highlight the importance of NEXN in the cardiovascular system and its potential role in regulating VSMCsʼ phenotypic transition. However, further in-depth investigation is necessary to elucidate the precise mechanisms by which NEXN modulates VSMCs and NEXNʼs involvement and causal relationships in vascular remodeling-related diseases.

Here, we demonstrate that NEXN is a critical molecule that maintains the contractile phenotype in VSMCs and regulates neointimal hyperplasia, as supported by integrative transcriptomics data analysis. NEXN expression is downregulated in both synthetic VSMCs and neointimal areas following injury. Using human aortic isolated SMCs (HASMCs), VSMC-specific NEXN-knockout mice, and the carotid artery wire injury model combined with RNA-Seq, we demonstrated that VSMC-derived NEXN mediates VSMCsʼ phenotypic switching and plays a mechanistic role in neointimal hyperplasia via endoplasmic reticulum (ER) stress–induced cell cycle progression. This study reveals an important causal role of NEXN in the uncontrolled proliferation of VSMCs and subsequent arterial disorders, suggesting NEXN as a potential therapeutic target for proliferative arterial diseases.

## Results

### Integrative transcriptomics data analysis identifies candidate genes potentially associated with VSMC phenotypic switching.

To identify candidate genes associated with the cellular heterogeneity of VSMCs during vascular injury, we analyzed single-cell transcriptomic data from injured mouse femoral arteries (GSE182232) (17). The dataset included samples from sham-operated mice and mice subjected to wire-induced injury for 2 or 4 weeks. After quality control, we obtained 24,962 cells and grouped them into 19 clusters through unsupervised clustering using Seurat (Supplemental Figure 1, A and B; supplemental material available online with this article; https://doi.org/10.1172/jci.insight.190089DS1), consistent with the original study (17). Among these, 4 clusters were identified as VSMCs, which were specially enriched for classical contractile genes including myosin heavy chain 11 (*MYH11*); actin alpha 2, smooth muscle (*ACTA2*); and transgelin (*TAGLN*) (Supplemental Figure 1B). These 4 clusters comprised 5,990 cells. We then calculated a contraction score for each single cell based on these contractile gene signatures.

**Figure 1:**
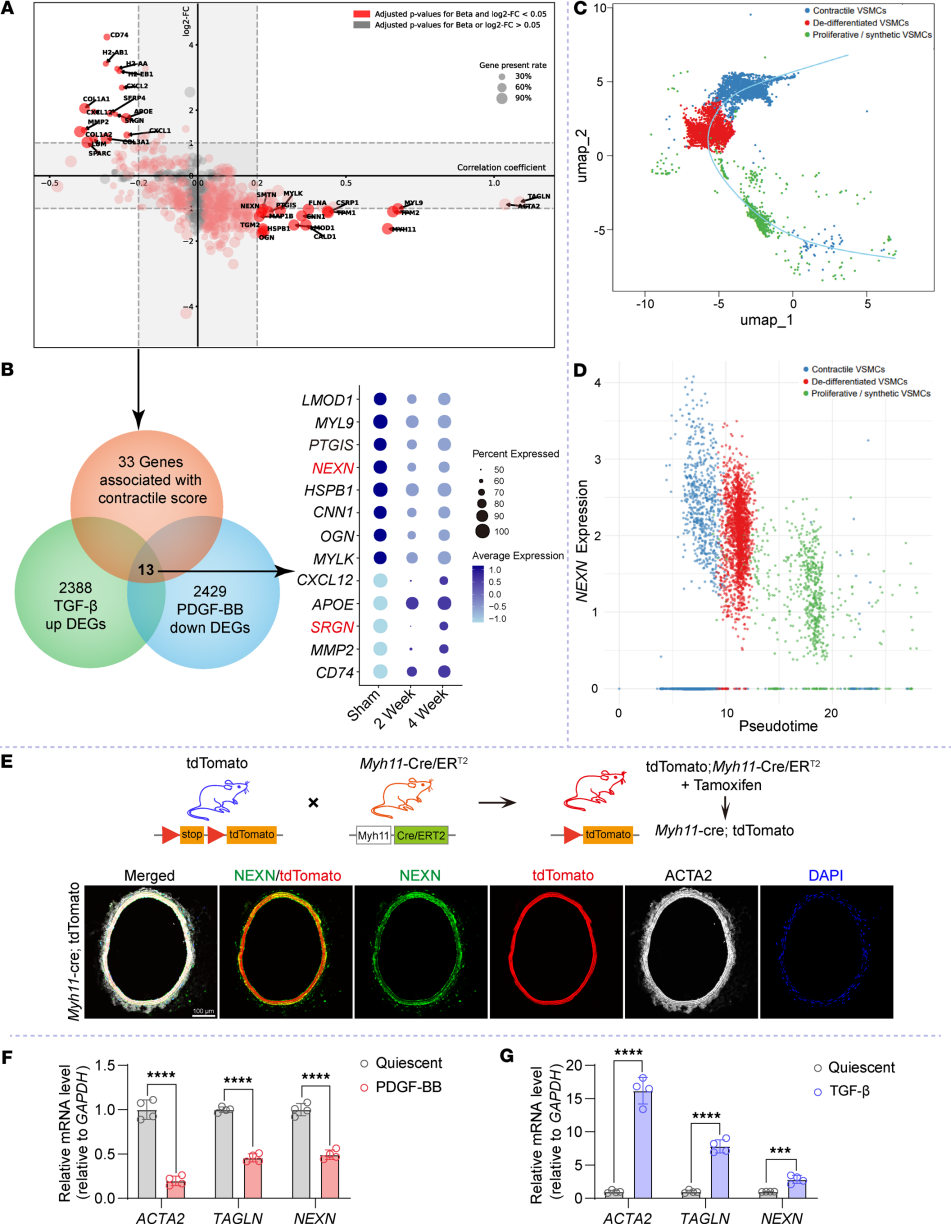
Integrative transcriptomics data analysis predicts candidate proteins associated with the VSMC phenotype. (**A**) Genes expressed in at least 20% of VSMCs, showing correlation coefficients with contraction scores and the log_2_(fold-change) (log_2_FC) after wire-induced femoral artery injury compared with sham-operated controls. Genes with adjusted *P* < 0.05 and correlation coefficients > 0.2 or < –0.2 are highlighted in red. (**B**) Left, Venn diagram illustrating the overlap of 13 candidate genes significantly correlated with contraction scores and differentially expressed in TGF-β–treated or PDGF-BB–treated rat VSMCs. Right, expression of these 13 candidate genes at different time points in femoral arteries (sham-operated, 2-week wire-induced injury, or 4-week wire-induced injury). DEGs, differentially expressed genes. (**C**) Trajectory analysis of reclustered VSMCs differentially enriched for contractile and synthetic markers as shown in Supplemental Figure 1C. (**D**) NEXN expression along the pseudotime trajectory of VSMC clusters. (**E**) Upper, strategy for generating *Myh11*-Cre/ER^T2^ R26-tdTomato mice. Lower, representative immunofluorescence images of NEXN (green), tdTomato (red), and ACTA2 (white) in the normal carotid artery of *Myh11*-Cre/ER^T2^ tdTomato mice. Scale bar: 100 μm. (**F** and **G**) Quantitative real-time PCR (qRT-PCR) was conducted to measure the mRNA levels of *NEXN*, *ACTA2*, and *TAGLN* in HASMCs treated with PDGF-BB (**F**) or TGF-β (**G**) for 24 hours. *n* = 4 for each group. Data are represented as mean ± SEM. Statistical analyses were performed using unpaired, 2-tailed Student’s *t* test. ***P < 0.001, ****P < 0.0001 for indicated comparisons.

We ranked 595 genes expressed in at least 20% of VSMCs by their correlation coefficients with the contraction score, estimated using linear mixed effects model adjusted for housekeeping genes and including mouse samples as a random effect term. Applying a stringent statistical threshold (adjusted *P* < 0.05 and correlation coefficients > 0.2 or < –0.2), we identified 31 genes positively correlated and 49 negatively correlated with contraction scores (Figure 1A). Among these, 17 positively correlated genes were significantly downregulated and 16 negatively correlated genes were upregulated after wire-induced injury of the femoral arteries compared with sham-operated controls (adjusted *P* < 0.05 and |log_2_FC| > 1; Figure 1A).

To further validate the changes in candidate genes associated with VSMCsʼ phenotypic switching, we compared our findings with differential gene expression data from TGF-β–treated and PDGF-BB–treated rat VSMCs provided in the study by Wei et al. (18). We found that 13 of out candidate genes overlapped with 2,388 upregulated and 2,429 downregulated genes from these treatments (Figure 1B). Among these 13 genes, *LMOD1*, *MYL9*, *PTGIS*, *HSPB1*, *CNN1*, *OGN*, *MYLK*, *CXCL12*, *APOE*, *MMP2*, and *CD74* are well-established markers associated with VSMC phenotypic switching. The remaining 2 genes, *NEXN* and *SRGN*, are less characterized in this context. To further characterize NEXN expression across VSMC lineages, we reclustered the VSMC population into 3 groups: contractile VSMCs, dedifferentiated VSMCs, and synthetic/proliferative VSMCs, based on their enrichment for contractile and synthetic gene signatures (Supplemental Figure 1C). Using the unsupervised inference method Monocle, we identified a clear directional trajectory where contractile VSMCs were positioned at the opposite end of the synthetic/proliferative clusters (Figure 1C). Along this trajectory, NEXN expression decreased from the quiescent contractile state toward the synthetic/proliferative state (Figure 1D), suggesting a strong association between NEXN expression and VSMC phenotypic switching.

To validate the localization and expression of NEXN mouse vessels, we found that NEXN was mainly localized to VSMCs within the media layer of mouse arteries using the *Myh11*-Cre/ER^T2^ *Rosa26*-tdTomato (*Myh11*-Cre/ER^T2^ tdTomato) lineage-tracing mice (Figure 1E). Subsequent quantitative reverse transcription PCR (qRT-PCR) analysis revealed a decrease in *NEXN* mRNA levels alongside VSMC contractile markers following PDGF-BB stimulation in HASMCs (Figure 1F). Conversely, HASMCs treated with TGF-β exhibited increased mRNA levels of both *NEXN* and contractile markers (Figure 1G). These integrative transcriptomics analyses and lineage-tracing results further support the role of NEXN as a critical regulatory factor in VSMC phenotypic transition.

### NEXN reduction correlates with VSMC phenotypic switching and neointimal hyperplasia.

**Figure 2:**
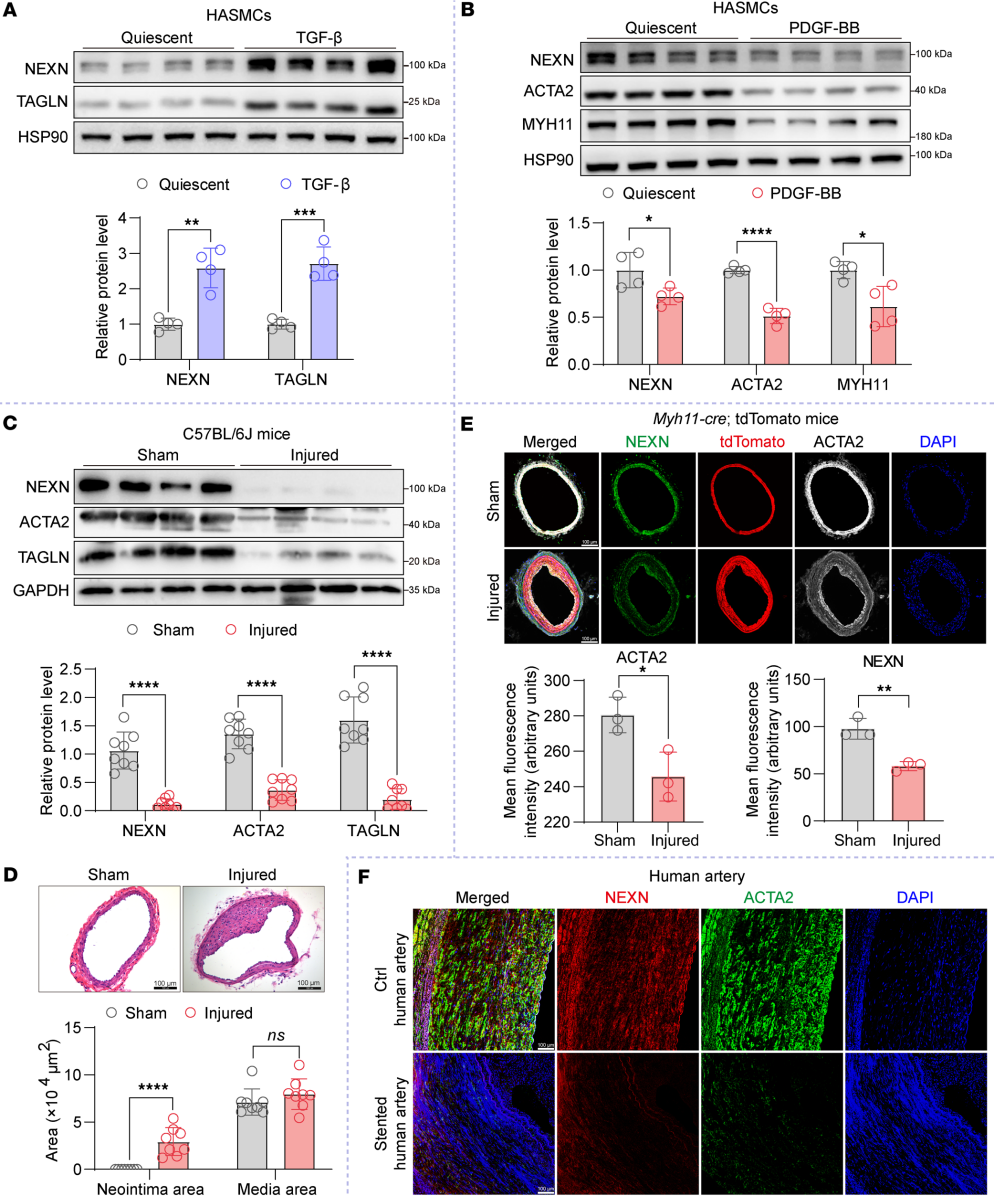
The expression of NEXN is significantly diminished during the processes of VSMC phenotypic switching and neointimal hyperplasia. (**A** and **B**) Immunoblotting and quantification of NEXN and the VSMC contractile proteins (ACTA2, TAGLN, and MYH11) in lysates of HASMCs treated with 10 ng/mL TGF-β for 24 hours (**A**) or 20 ng/mL PDGF-BB for 24 hours (**B**). *n* = 4 for each group. (**C**) Representative Western blotting and quantification of NEXN and the VSMC contractile proteins (ACTA2 and TAGLN) in the lysates extracted from sham-operated or wire-injured carotid arteries of C57BL/6J mice. *n* = 8 for each group. (**D**) Upper, representative cross sections of H&E-stained sham-operated and wire-injured carotid arteries from C57BJ/6L mice. Scale bar: 100 μm. Lower, quantitative analysis of the neointima area and media area in histological staining sections. *n* = 8 for each group. (**E**) Representative immunofluorescence images and quantification of NEXN (green), tdTomato (red), and ACTA2 (white) in the sham or injured carotid arteries of *Myh11*-CreER^T2^ tdTomato mice. Scale bar: 100 μm. *n* = 3 for each group. (**F**) Representative immunofluorescence images of NEXN (red) and ACTA2 (green) in control (Ctrl) human artery and stented human artery. Scale bar: 100 μm. Data are represented as mean ± SEM. Statistical analyses were performed using unpaired, 2-tailed Student’s *t* tests. *P < 0.05, **P < 0.01, ***P < 0.001, ****P < 0.0001 for indicated comparisons.

Western blot analysis revealed an increase in NEXN protein levels in HASMCs treated with TGF-β (Figure 2A). In contrast, PDGF-BB treatment significantly downregulated NEXN expression, consistent with the transdifferentiation status of VSMCs and the observed changes in contractile marker expression (Figure 2B). Notably, we verified a pronounced reduction in NEXN protein levels in mouse carotid arteries at 28 days following wire-induced injury compared with sham-operated arteries. This reduction was associated with decreased expression of contractile markers in VSMCs and an expansion of neointimal areas (Figure 2, C and D). To investigate our understanding of cellular alterations of NEXN during intimal hyperplasia following vascular injury, we utilized VSMC lineage-tracing *Myh11*-Cre/ER^T2^ tdTomato mice to establish a carotid artery guide wire injury model. Immunostaining of NEXN, ACTA2, and tdTomato in the carotid arteries of *Myh11*-Cre/ER^T2^ tdTomato mice revealed that, in the injured carotid arteries, tdTomato positivity and ACTA2 low-expression regions consisted of synthetic VSMCs, which exhibited lower NEXN expression levels compared with uninjured carotid arteries (Figure 2E). Consistently, immunofluorescence analysis revealed a substantial decrease in NEXN expression in stented human arteries compared with control human arteries (Figure 2F). These findings suggest that downregulation of NEXN is associated with VSMC phenotypic switching and contributes to neointimal hyperplasia.

### NEXN maintains the contractile phenotype of VSMCs in vitro.

**Figure 3:**
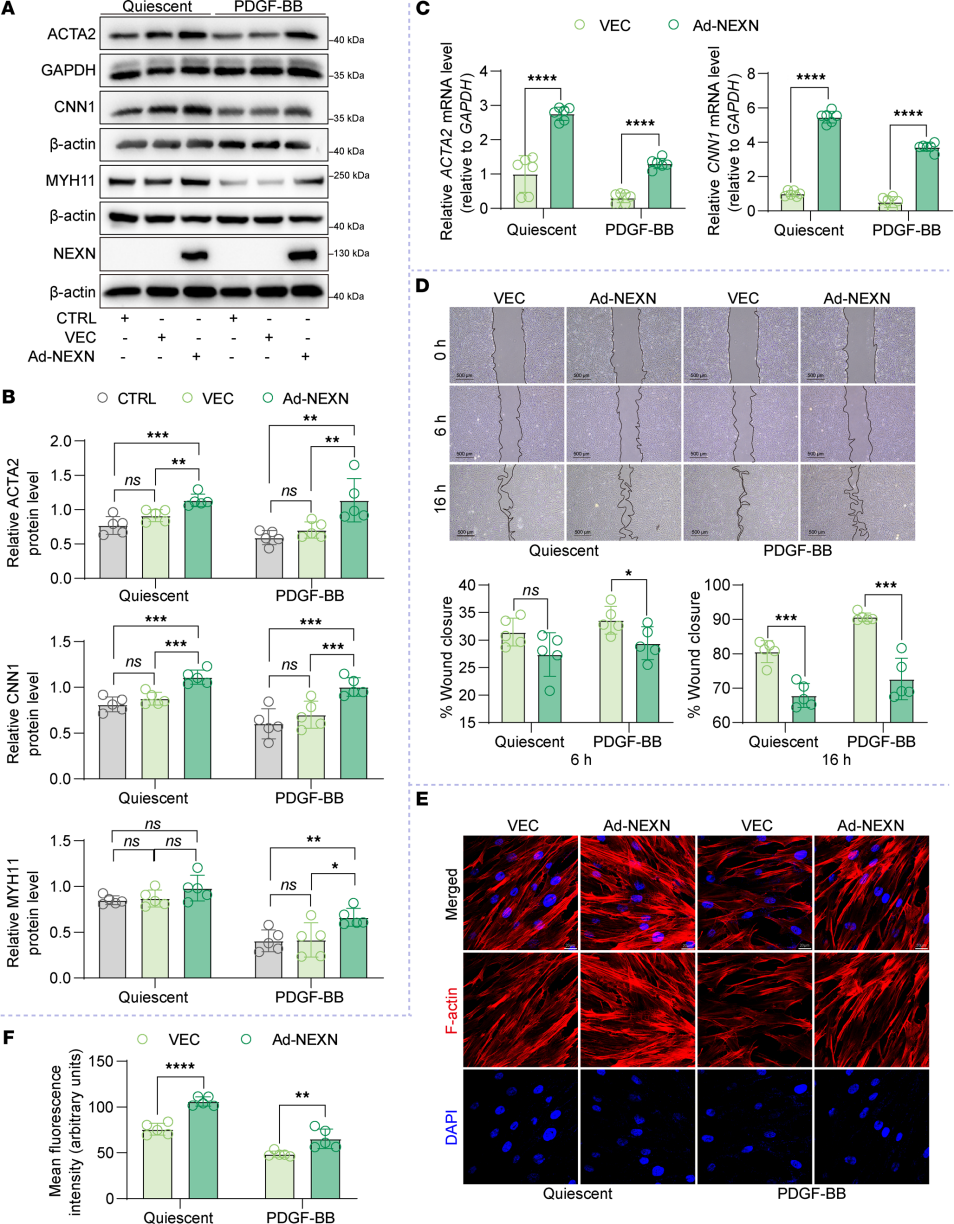
NEXN maintains the contractile phenotype of VSMCs in vitro. (**A** and **B**) Immunoblotting (**A**) and quantification (**B**) of the VSMC contractile proteins (ACTA2, CNN1, and MYH11) and NEXN in lysates of HASMCs preinfected with CTRL (control), VEC (vector), or NEXN adenovirus (Ad-NEXN) treated with PDGF-BB for 24 hours. *n* = 5 for each group. (**C**) qRT-PCR analysis of mRNA levels of VSMC contractile marker (*ACTA2* and *CNN1*) in VEC- or Ad-NEXN–preinfected HASMCs treated with PDGF-BB for 24 hours. *n* = 6 for each group. (**D**) Representative images and quantitative analysis of scratch wound–healing analysis for HASMCs preinfected with VEC or Ad-NEXN stimulated with PDGF-BB for 24 hours. Images were taken at 0, 6, and 16 hours after scratching and PDGF-BB stimulating. Scale bar: 500 μm. *n* = 5 for each group. (**E**) Representative immunofluorescence images and (**F**) quantification of F-actin (red) stained with phalloidin in HASMCs preinfected with VEC or Ad-NEXN for 48 hours followed by PDGF-BB treatment for another 24 hours. Scale bar: 20 μm. *n* = 5 for each group. Data are represented as mean ± SEM. Statistical analyses were performed using unpaired, 2-tailed Student’s *t* tests (**C**, **D**, and **F**) or 1-way ANOVA (**B**). *P < 0.05, **P < 0.01, ***P < 0.001, ****P < 0.0001 for indicated comparisons.

VSMCs exhibit significant phenotypic modulation, ranging from a contractile state in quiescent mature arteries to a proliferative and synthetic state in neointimal hyperplasia. To investigate the effects of elevated NEXN levels on VSMC phenotype, we employed NEXN adenovirus to overexpress NEXN in HASMCs (Supplemental Figure 2). NEXN overexpression attenuated PDGF-BB–induced VSMC phenotypic switching, as evidenced by an increase in the mRNA and protein levels of contractile markers (Figure 3, A–C). In a scratch wound–healing assay, NEXN overexpression significantly inhibited the migration of VSMCs, both in resting and in PDGF-BB–stimulated HASMCs (Figure 3D). F-actin staining revealed that NEXN overexpression increased F-actin levels in resting VSMCs. It also counteracted the decrease in F-actin levels caused by PDGF-BB stimulation, thus maintaining the contractile phenotype (Figure 3, E and F). These findings indicate that increased NEXN plays a protective role in maintaining the contractile phenotype of VSMCs.

### NEXN silencing exacerbates the phenotypic switching of VSMCs in vitro.

**Figure 4:**
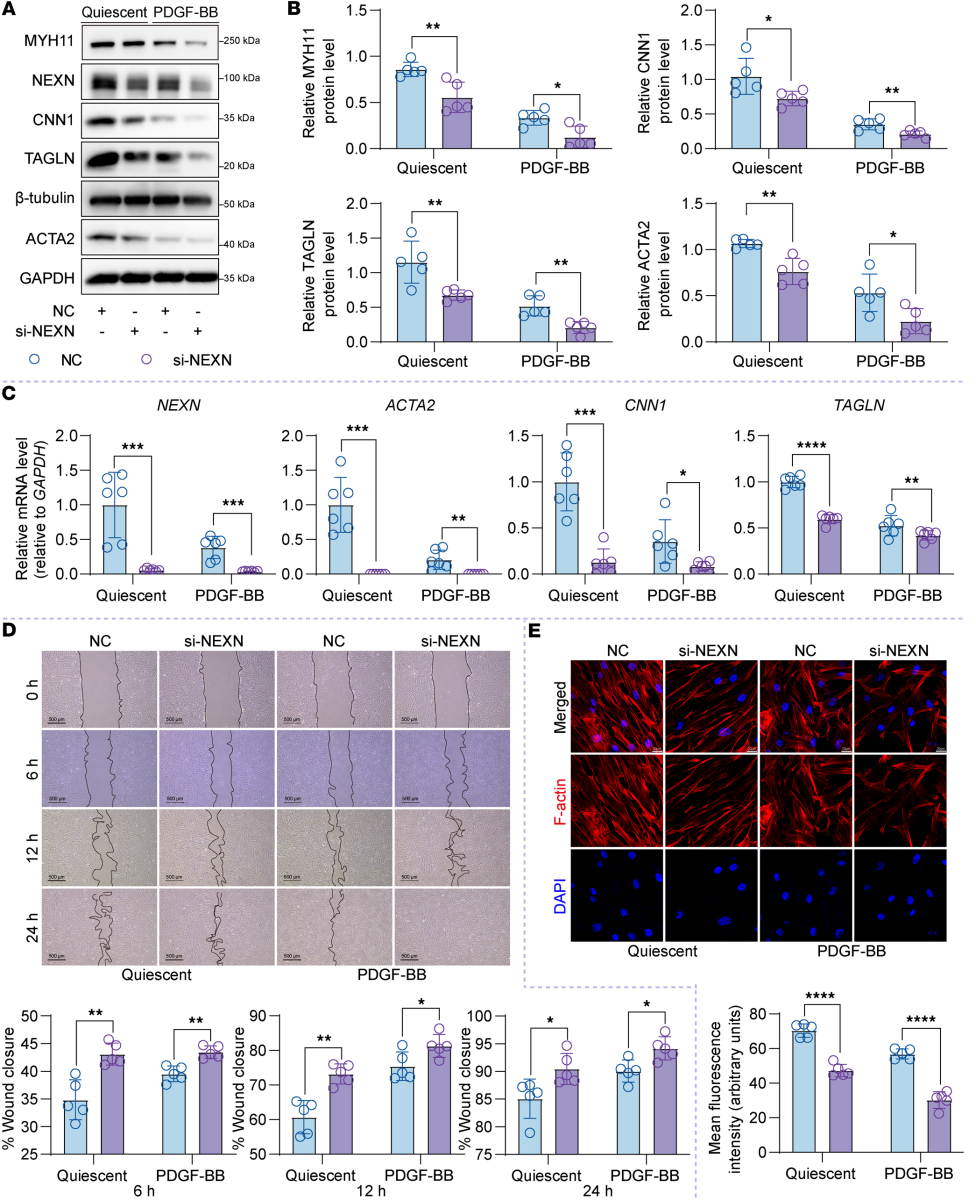
NEXN knockdown in VSMCs aggravates VSMCsʼ phenotypic switching in vitro. (**A** and **B**) Immunoblotting (**A**) and quantification (**B**) of the VSMC contractile proteins (MYH11, CNN1, TAGLN, and ACTA2) in lysates of HASMCs pretransfected with NC (negative control) or NEXN siRNA (si-NEXN) treated with PDGF-BB for 24 hours. *n* = 5 for each group. (**C**) qRT-PCR analysis of mRNA levels of VSMC contractile markers (*MYH11*, *CNN1*, *TAGLN*, and *ACTA2*) in NC- or si-NEXN–preinfected HASMCs treated with PDGF-BB for 24 hours. *n* = 5 for each group. (**D**) Representative images and quantitative analysis of scratch wound–healing analysis for HASMCs pretransfected with NC or si-NEXN stimulated with PDGF-BB for 24 hours. Images were taken at 0, 6, 12, and 24 hours after scratching and PDGF-BB stimulating. Scale bar: 500 μm. *n* = 5 for each group. (**E**) Representative immunofluorescence images and quantification of F-actin (red) stained with phalloidin in HASMCs transfected with NC or si-NEXN for 48 hours followed by PDGF-BB for another 24 hours. Scale bar: 20 μm. *n* = 5 for each group. Data are represented as mean ± SEM. Statistical analyses were performed using unpaired, 2-tailed Student’s *t* tests. *P < 0.05, **P < 0.01, ***P < 0.001, ****P < 0.0001 for indicated comparisons.

Next, we investigated the effects of NEXN silencing on VSMC phenotypic transition using small interfering RNA (siRNA) targeting NEXN (Supplemental Figure 3). Knockdown of NEXN induced a switch from a contractile to a synthetic phenotype in both resting and PDGF-BB–stimulated HASMCs, as evidenced by decreased expression of contractile phenotype markers, such as MYH11, ACTA2, CNN1, and TAGLN (Figure 4, A–C). Meanwhile, in vitro scratch assays were performed to investigate the role of NEXN in VSMC migration in response to PDGF-BB. Our results showed that NEXN deletion enhanced the migration of VSMCs induced by PDGF-BB (Figure 4D). F-actin staining demonstrated that silencing NEXN reduced F-actin levels and promoted a phenotypic switch in VSMCs from an elongated contractile phenotype to a polygonal synthetic phenotype (Figure 4E). Collectively, these findings suggest that NEXN is both necessary and sufficient for maintaining the contractile phenotype of VSMCs in vitro.

### NEXN deficiency in VSMCs exacerbates postinjury neointima formation in vivo.

**Figure 5:**
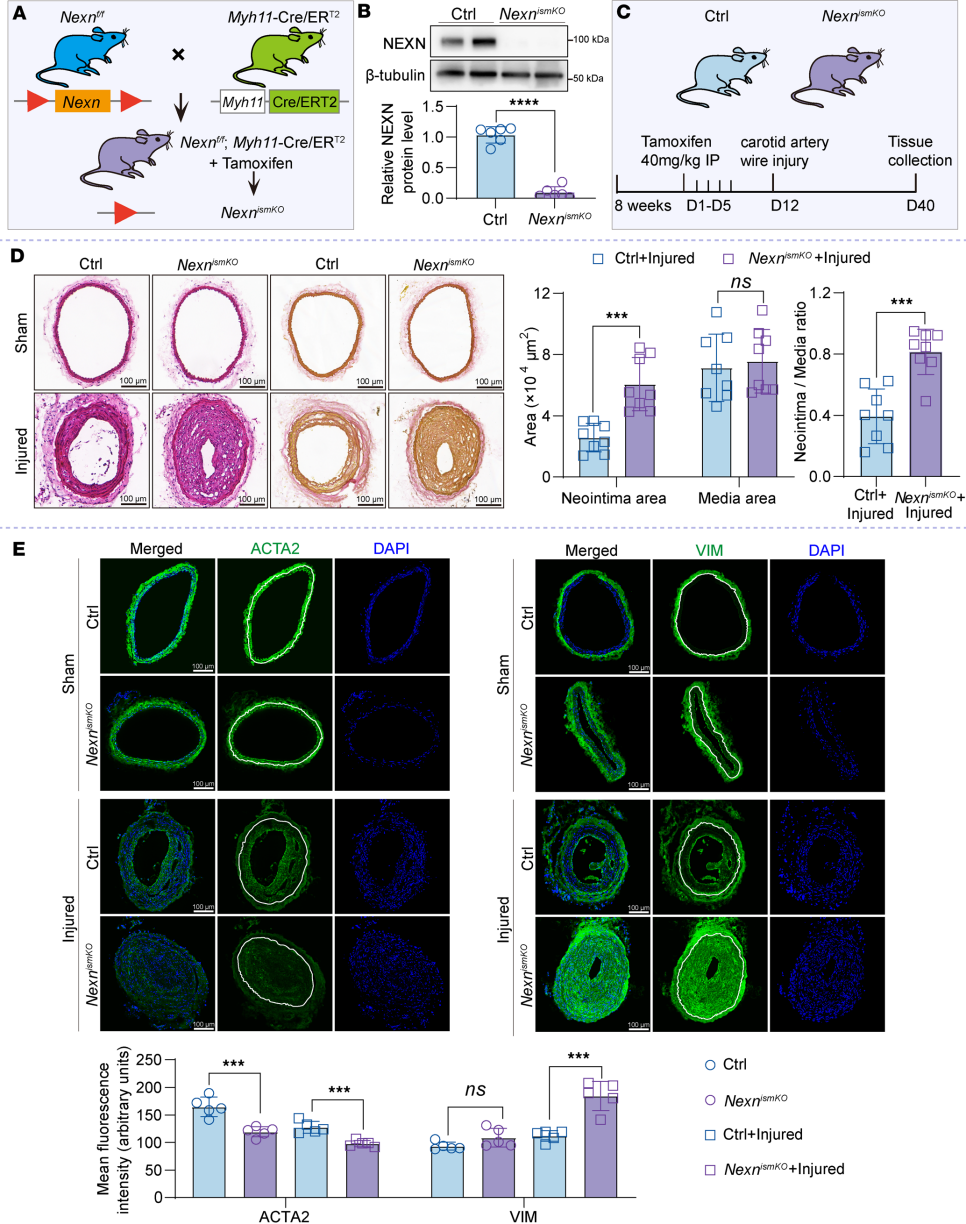
VSMC-specific deletion of NEXN aggravates neointima formation following vascular injury in mice. (**A**) Strategy for generating *Nexn^ismKO^* mice by crossing *Nexn^fl/fl^* mice with *Myh11*-Cre/ER^T2^ mice. (**B**) Immunoblotting and quantification of the knockout efficiency of NEXN in the aorta of Ctrl and *Nexn^ismKO^* mice. *n* = 6 for each group. (**C**) Schematic timeline of tamoxifen treatment and carotid artery wire injury model. (**D**) Left, representative cross sections of H&E- and VVG-stained sham-operated and wire-injured carotid arteries from the Ctrl and *Nexn^ismKO^* mice. Scale bar: 100 μm. Right, quantitative analysis of the neointima area, media area, and neointima area to medial area ratio in histological staining sections. *n* = 8 for each group. (**E**) Immunostaining and quantification of the VSMC contractile protein (ACTA2) and synthetic protein (vimentin, VIM) on sections of uninjured right common carotid artery or injured left common carotid artery from the Ctrl and *Nexn^ismKO^* mice. Scale bar: 100 μm. *n* = 5 for each group. Data are represented as mean ± SEM. Statistical analyses were performed using unpaired, 2-tailed Student’s *t* tests. ***P < 0.001, ****P < 0.0001 for indicated comparisons.

To further evaluate the involvement of NEXN in VSMC phenotypic transition and subsequent neointima formation in vivo, *Nexn*-floxed (*Nexn^fl/fl^*) mice were generated and interbred with *Myh11*-Cre/ER^T2^ mice to produce *Nexn* smooth muscle–specific knockout mice with tamoxifen-inducible deletion, termed *Nexn^ismKO^* mice (Figure 5A). Western blot analysis confirmed that Nexn expression in the aorta was specifically eliminated in *Nexn^ismKO^* mice compared with Ctrl mice (Figure 5B).

To investigate the impact of *Nexn* deficiency on arterial neointima formation, *Nexn^ismKO^* and Ctrl mice were subjected to left common carotid artery wire injury following tamoxifen administration. The right common carotid artery served as the sham operation group, undergoing no surgical intervention. The common carotid arteries were harvested 28 days after surgery (Figure 5C). Hematoxylin-eosin (H&E) and Verhoeff–Van Gieson (VVG) staining of serial cross sections of the left injured carotid artery and the right uninjured carotid artery revealed a significantly increased neointima area and neointima/media ratio in *Nexn^ismKO^* mice compared with Ctrl mice (Figure 5D). However, there was no difference in the media area between *Nexn^ismKO^* mice and Ctrl mice (Figure 5D). Immunostaining showed a reduction in the contractile protein ACTA2 and an increase in the synthetic protein VIM in the medial and neointimal layers of carotid arteries from *Nexn^ismKO^* mice compared with those from Ctrl mice (Figure 5E). These findings are consistent with the in vitro data, indicating that VSMC-specific deletion of NEXN in mice exacerbates the development and severity of postinjury neointima formation.

### NEXN is primarily involved in regulating ER stress and cell cycle progression during phenotypic switching of VSMCs.

**Figure 6:**
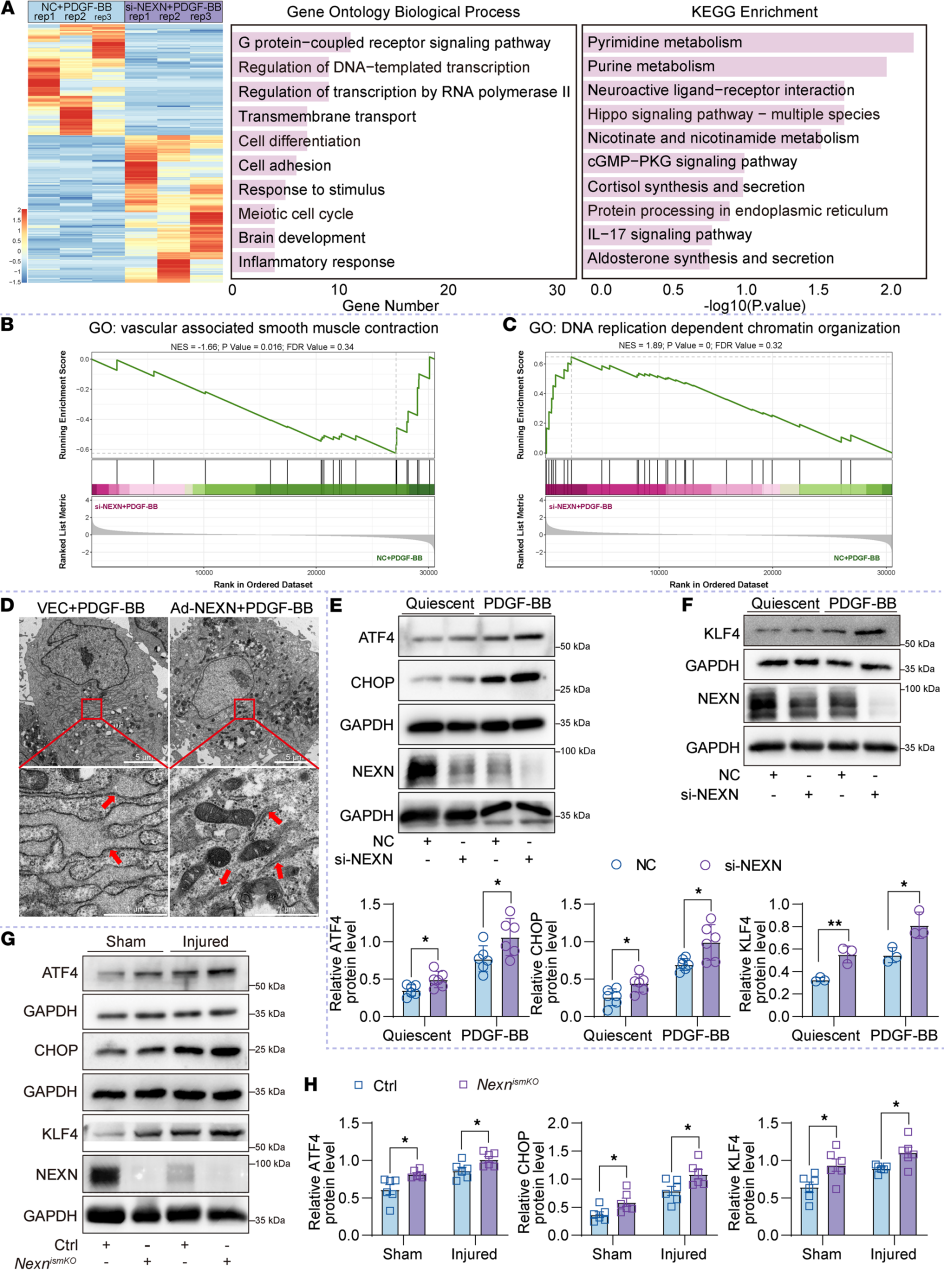
NEXN knockdown in VSMCs augments ER stress, modulating the VSMC phenotype via the ATF4/Krüppel-like factor 4 signaling axis. (**A**) GOBPs and KEGG enrichment analysis of DEGs in NC- or si-NEXN–pretransfected HASMCs treated with PDGF-BB. *n* = 3 for each group. (**B**) GSEA-based GO analysis enrichment plots of vascular associated smooth muscle contraction. (**C**) GSEA-based GO analysis enrichment plots of DNA replication-dependent chromatin organization. (**D**) Transmission electron microscopy images of VEC- or Ad-NEXN–preinfected HASMCs treated with PDGF-BB. Arrows indicate the ER. Scale bar: top = 5 μm; bottom = 1 μm. (**E**) Immunoblotting and quantification of ER stress indicators (ATF4 and CHOP) in lysates of NC or si-NEXN pretransfected HASMCs treated with PDGF-BB for 24 hours. *n* = 6 for each group. KLF4, Krüppel-like factor 4. (**F**) Immunoblotting and quantification of KLF4 in lysates of NC- or si-NEXN–pretransfected HASMCs treated with PDGF-BB for 24 hours. *n* = 3 for each group. (**G** and **H**) Immunoblotting (**G**) and quantification (**H**) of ER stress indicators (ATF4 and CHOP) and KLF4 expression in lysates prepared from either sham-operated right common carotid arteries or injured left common carotid arteries of Ctrl and *Nexn^ismKO^* mice. *n* = 6 for each group. Data are represented as mean ± SEM. Statistical analyses were performed using unpaired, 2-tailed Student’s *t* tests. *P < 0.05, **P < 0.01 for indicated comparisons.

To elucidate the role of NEXN in VSMC phenotypic switching and postinjury neointima formation, we performed bulk RNA-Seq of PDGF-BB–stimulated HASMCs transfected with NC and NEXN siRNA. The high quality of the RNA-Seq was evidenced by the reproducibility between independent experiments (Supplemental Figure 4A). DEGs were identified with a |log_2_FC|>1 (FC > 2 or < 0.5) and a *P* < 0.05. Our research identified significant alterations in gene expression profiles between si-NEXN+PDGF-BB and NC+PDGF-BB HASMCs. Specifically, 150 genes were upregulated, while 197 genes were downregulated in si-NEXN+PDGF-BB HASMCs compared with NC+PDGF-BB (Supplemental Figure 4B). Functional annotation of DEGs was performed using gene ontology biological processes (GOBPs) analysis and Kyoto Encyclopedia of Genes and Genomes (KEGG) pathway analysis. GOBPs analysis revealed that the DEGs were primarily enriched in the following biological processes: GPCR signaling pathway, regulation of DNA-templated transcription, cell differentiation, and cell cycle (Figure 6A). KEGG analysis indicated significant enrichment of DEGs in pyrimidine metabolism, purine metabolism, the Hippo signaling pathway, and protein processing in the ER (Figure 6A). To further investigate the molecular mechanisms by which NEXN regulates the phenotypic switching of VSMCs, we performed the overall analysis for gene set enrichment analysis–based (GSEA-based) GO analysis (Supplemental Table 6). Among these, DNA replication-dependent chromatin organization plays a crucial role in regulating the phenotype of VSMCs (19, 20). Figure 6, B and C, revealed that interfering with NEXN significantly inhibited vascular associated smooth muscle contraction while promoting DNA replication-dependent chromatin organization in PDGF-BB–stimulated HASMCs, compared with the NC group. The ER occupies a unique position because of its proximity to the nucleus, which is essential for coordinating cellular events, such as protein synthesis and cell cycle processes (21–23). The cell cycle is dispensable for the migration and proliferation of VSMCs involved in proliferative vascular diseases such as in-stent restenosis and atherosclerosis. Based on unbiased RNA-Seq data and our previous studies, we hypothesize that NEXN deficiency induces ER stress, leading to enhanced proliferation of VSMCs and exacerbating the phenotypic switch that contributes to intimal hyperplasia.

ER stress is characterized by increased expression of ER stress markers and abnormal ER morphology. To further elucidate the impact of NEXN on ER function, we employed transmission electron microscopy (TEM) to examine the morphology of PDGF-BB–stimulated HASMCs transfected with VEC and NEXN adenoviruses. TEM revealed markedly enhanced ER stress in PDGF-BB–stimulated HASMCs, as evidenced by more extensive and abundant expansion of the ER membrane. Overexpression of NEXN markedly attenuated PDGF-BB–induced ER stress, resulting in reduced ER expansion, diminished membrane vesicles, and improved membrane integrity (Figure 6D). We also analyzed ER stress signaling in both resting and PDGF-BB–stimulated HASMCs using immunoblotting. Our findings revealed that NEXN knockdown significantly elevated the levels of ER stress indicators activating transcription factor 4 (ATF4) and DNA-damage inducible transcript 3 (CHOP, also known as DDIT3) (Figure 6E). Kaw et al. demonstrated that augmented ER stress modulates VSMCsʼ phenotype via the ATF4/KLF4 signaling axis (24). Correspondingly, we also found that interfering with NEXN could significantly increase the expression of KLF4 in resting conditions and during PDGF-BB stimulation in HASMCs (Figure 6F). Furthermore, we validated the role of NEXN in regulating VSMCsʼ phenotypic switching via the ATF4/KLF4 signaling axis in the carotid artery wire injury model using NEXN VSMC-specific knockout and control mice. Our results demonstrate that VSMC-specific deletion of NEXN significantly elevated the levels of ER stress markers, including ATF4 and CHOP, as well as the expression of KLF4, in both sham-operated and injured common carotid arteries (Figure 6, G and H). These findings provide strong in vivo evidence supporting the molecular mechanisms by which NEXN regulates VSMCsʼ phenotypic switching and postinjury neointima formation via the ATF4/KLF4 signaling axis.

**Figure 7:**
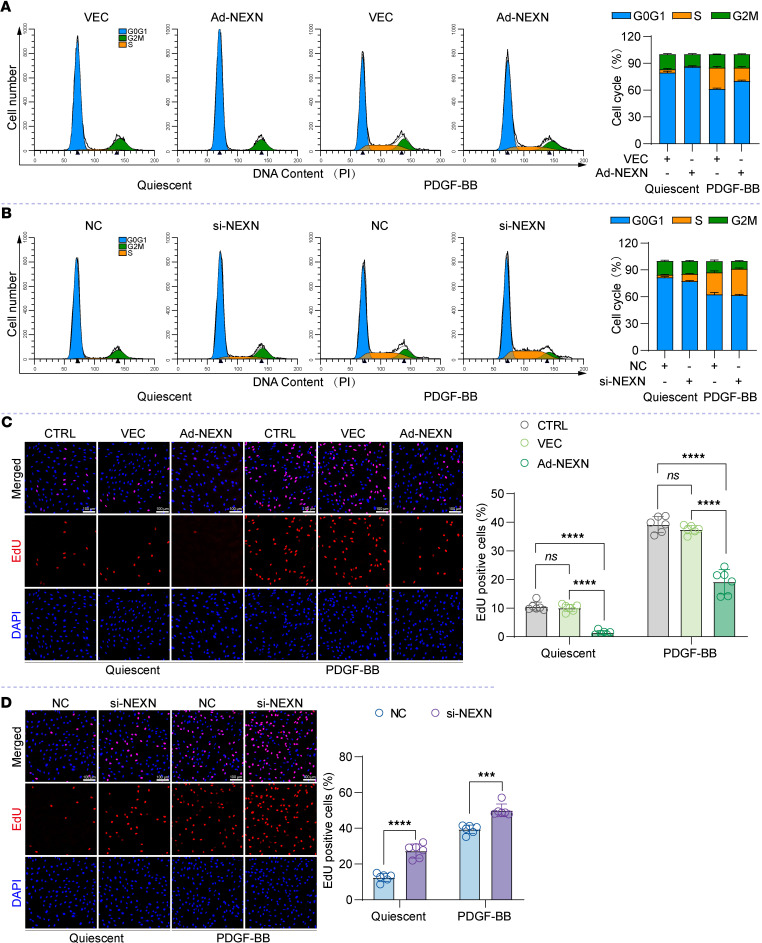
NEXN is primarily responsible for regulating cell cycle progression and cell proliferation. (**A**) Flow cytometry analysis of cell cycle transition in VEC- or Ad-NEXN–preinfected HASMCs treated with PDGF-BB for 24 hours. *n* = 6 for each group. (**B**) Flow cytometry analysis of cell cycle transition in NC- or si-NEXN–pretransfected HASMCs treated with PDGF-BB for 24 hours. *n* = 6 for each group. (**C**) Representative EdU staining and quantification in CTRL-, VEC-, or Ad-NEXN–preinfected HASMCs treated with PDGF-BB for 24 hours. Scale bar: 100 μm. *n* = 6 for each group. (**D**) Representative EdU staining and quantification in NC- or si-NEXN–pretransfected HASMCs treated with PDGF-BB for 24 hours. Scale bar: 100 μm. *n* = 6 for each group. Data are represented as mean ± SEM. Statistical analyses were performed using unpaired, 2-tailed Student’s *t* tests (**D**) or 1-way ANOVA (**C**). ***P < 0.001, ****P < 0.0001 for indicated comparisons.

We used flow cytometry to investigate the effect of NEXN on the cell cycle of VSMCs. The results demonstrated that NEXN overexpression (Ad-NEXN) markedly reduced the S phase cell population in both resting and PDGF-BB–stimulated HASMCs compared with the VEC group (Figure 7A). Conversely, NEXN knockdown (si-NEXN) led to the accumulation of cell populations in S phase, accompanied by a decrease in the G0/G1 population compared with the NC group (Figure 7B). The precise transition from the G1 phase to the S phase of the cell cycle is crucial for regulating eukaryotic cell proliferation. To determine whether NEXN regulates VSMCs proliferation, we performed 5-ethynyl-2′-deoxyuridine (EdU) cell proliferation assays. NEXN overexpression reduced the proliferation of both quiescent and PDGF-BB–stimulated HASMCs (Figure 7C). In contrast, NEXN knockdown increased the percentage of EdU-positive cells in both quiescent and PDGF-BB–treated HASMCs (Figure 7D). These data provide compelling evidence that NEXN plays a key role in regulating ER stress and cell cycle progression during phenotypic switching of VSMCs.

### NEXN modulates the phenotypic transition and proliferation of VSMCs through ER stress.

**Figure 8:**
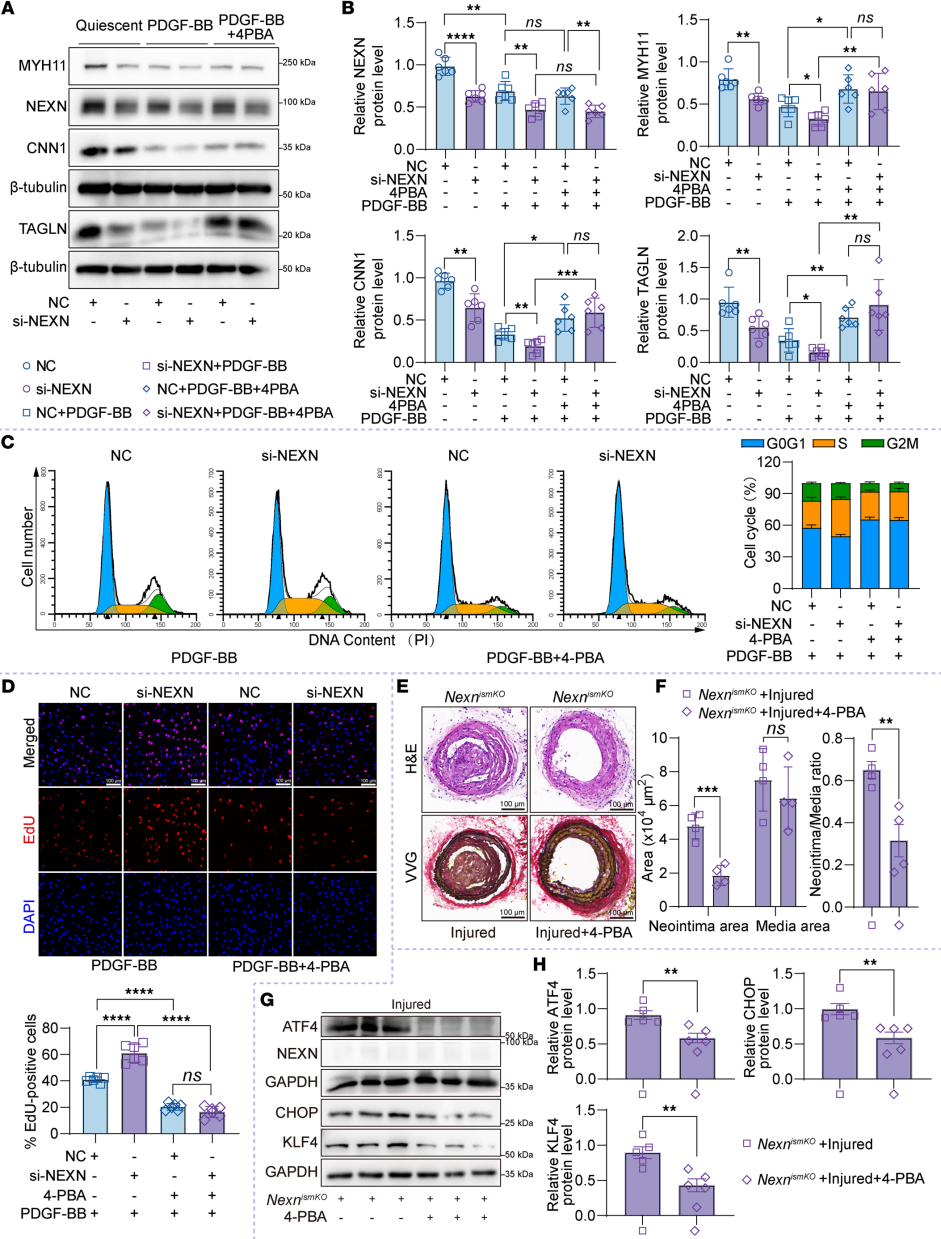
Inhibiting ER stress could ameliorate VSMC proliferation and phenotypic transition induced by NEXN deficiency. (**A** and **B**) Immunoblotting (**A**) and quantification (**B**) of NEXN and the VSMC contractile proteins (MYH11, CNN1, TAGLN) expression in lysates of NC- or si-NEXN–pretransfected HASMCs treated with PDGF-BB (20 ng/mL) and 4-PBA (2.5 mM) for 24 hours. (**C**) Flow cytometry analysis of cell cycle transition in NC- or si-NEXN–pretransfected HASMCs treated with PDGF-BB and 4-PBA for 24 hours. *n* = 6 for each group. (**D**) Representative EdU staining and quantification in NC- or si-NEXN–pretransfected HASMCs treated with PDGF-BB and 4-PBA for 24 hours. Scale bar: 100 μm. *n* = 6 for each group. (**E**–**H**) *Nexn^ismKO^* mice were subjected to a carotid artery wire injury model for a duration of 4 weeks. Throughout this period, the mice were randomly assigned to receive either saline or 4-PBA at a dosage of 20 mg/kg/d via injection. (**E**) Representative cross sections of H&E- and VVG-stained wire-injured carotid arteries from the *Nexn^ismKO^* mice with or without 4-PBA administration. Scale bar: 100 μm. (**F**) Quantitative analysis of the neointima area, media area, and neointima area to medial area ratio in histological staining sections depicted in **E**. *n* = 4 for each group. (**G** and **H**) Immunoblotting (**G**) and quantification (**H**) of ER stress indicators (ATF4 and CHOP) and KLF4 expression in lysates from injured left common carotid arteries of *Nexn^ismKO^* mice with or without 4-PBA administration. *n* = 6 for each group. Data are represented as mean ± SEM. Statistical analyses were performed using unpaired, 2-tailed Student’s *t* tests (**F** and **H**) or 1-way ANOVA (**B** and **D**). *P < 0.05, **P < 0.01, ***P < 0.001, ****P < 0.0001 for indicated comparisons.

To verify the role of NEXN in regulating VSMCsʼ phenotypic transition and vascular neointimal hyperplasia through ER stress, we utilized 4-phenylbutyric acid (4-PBA), an inhibitor of ER stress, to treat PDGF-BB–stimulated HASMCs that were transfected with NC and NEXN siRNA. The treatment with 4-PBA effectively prevented the phenotypic transition of VSMCs, as demonstrated by the increased expression of contractile protein markers in both the NC and si-NEXN groups in PDGF-BB–stimulated HASMCs (Figure 8, A and B). Flow cytometry analysis demonstrated 4-PBA treatment markedly reduced the accumulation of cell populations in the S phase of PDGF-BB–treated HASMCs triggered by NEXN silencing (Figure 8C). Correspondingly, the elevated EdU-positive cells in PDGF-BB–treated HASMCs after NEXN knockdown were significantly suppressed by 4-PBA treatment (Figure 8D). To further investigate the role of ER stress in NEXN-mediated neointima formation, we treated *Nexn^ismKO^* mice with the ER stress inhibitor 4-PBA following carotid artery wire injury. H&E and VVG staining revealed that 4-PBA treatment significantly reduced wire injury–induced neointima formation in carotid arteries from *Nexn^ismKO^* mice, as evidenced by a decrease in both neointima area and neointima/media ratio (Figure 8, E and F). Consistently, the increased expression of ATF4, CHOP, and KLF4 in postinjury carotid arteries induced by NEXN knockout was significantly inhibited by 4-PBA treatment (Figure 8, G and H). Collectively, these findings strongly suggest that dysregulated ER stress is pivotal for the uncontrolled VSMC proliferation and vascular neointimal hyperplasia induced by NEXN deficiency. Targeting ER stress is thus a potential therapeutic strategy for NEXN-related vascular proliferative diseases.

## Discussion

Phenotypic switch of VSMCs is a characteristic feature of cardiovascular diseases, including atherosclerosis and neointimal hyperplasia (25, 26). VSMCs exhibit remarkable heterogeneity in their response to these pathological stimuli. Emerging techniques like single-cell RNA sequencing (scRNA-Seq) provide a genome-wide profiling of individual cells, offering an unbiased approach to uncover cellular heterogeneity. In this study, we identify NEXN as a pivotal gene essential for phenotypic switching through integrative analysis of scRNA-Seq data from a vascular injury model and RNA-Seq data from TGF-β– and PDGF-BB–treated rat VSMCs.

Emerging genetic evidence has identified NEXN as a high-confidence gene associated with dilated cardiomyopathy (DCM), supporting its routine inclusion in the genetic evaluation of the condition (27, 28). Our previous research demonstrated NEXN as a component of junctional membrane complexes and provided mechanistic insights into how NEXN deficiency leads to the pathogenesis of severe DCM (11–14). Additionally, the association between NEXN and human coronary artery disease has been previously reported (16, 29). However, a comprehensive understanding of the cell-specific roles and precise mechanisms of NEXN in vascular remodeling remains lacking. Here, we demonstrated NEXN is highly expressed in VSMCs within the middle layer of arteries using the inducible lineage-tracing *Myh11*-Cre/ER^T2^ tdTomato mouse vascular injury model and human arteries with bare metal stents. Notably, NEXN expression is significantly downregulated during VSMC phenotypic transition and vascular injury-induced intimal hyperplasia.

To date, some research on NEXN in VSMCs has been largely limited to in vitro studies and non-VSMC-specific interventions in vivo (15, 16, 30). However, no studies have yet investigated how the absence of NEXN influences VSMC phenotypic transition and the development of neointimal hyperplasia. A particular strength of our study is that we generated VSMC-specific knockout mice to provide conclusive evidence that NEXN deficiency promotes the phenotypic transition of VSMCs and exacerbates the progress of neointimal hyperplasia.

Zhu et al. reported that NEXN expression is transcriptionally controlled by YAP and myocardin family coactivators in human coronary artery SMCs (15). Another study found that NEXN-AS1, a long noncoding RNA, could regulate the expression of NEXN in endothelial cells during atherosclerosis (29). However, there is still limited understanding of the downstream mechanism through which NEXN modulates the phenotypic switch in VSMCs, which is a key process in vascular remodeling, particularly in conditions like intimal hyperplasia. By combining RNA-Seq analysis with a series of experiments, we demonstrated that NEXN deficiency promotes cell cycle progression and proliferation through ER stress/KLF4 signaling, modulates VSMC phenotypic transition, and contributes to neointimal hyperplasia. These findings suggest that targeting NEXN could be a promising therapeutic strategy to prevent or mitigate neointimal hyperplasia, particularly in the context of restenosis following angioplasty.

This study focuses on ER-related functions primarily based on our previous findings in cardiomyocytes, where we observed that NEXN is predominantly localized at the contact sites between the plasma membrane and the ER/sarcoplasmic reticulum. NEXN modulates excitation-contraction coupling in cardiomyocytes by influencing ER calcium homeostasis (11, 12). Additionally, studies have demonstrated that blocking PERK/ATF4 ER stress signaling protects against the progression of aortic aneurysms (31). In light of these findings, this study demonstrates that NEXN deficiency promotes cell cycle progression and proliferation through the ER stress/KLF4 signaling axis, thereby modulating VSMC phenotypic transition and contributing to neointimal hyperplasia.

Notably, our transcriptomic data revealed enrichment of several other signaling pathways, such as G protein signaling, regulation of DNA-templated transcription, cell cycle–related signaling, pyrimidine metabolism, and purine metabolism. This suggests that NEXN may regulate VSMC phenotypic transition through a complex signaling network. It has been reported that prostacyclin and thromboxane signaling through GPCRs play a critical role in the phenotypic transition of VSMCs (32, 33). Recent studies have further identified that GPCR5B, which is highly expressed in aortic SMCs, controls smooth muscle contractility and differentiation by inhibiting prostacyclin receptor signaling (34). A limitation of this study is the lack of a systematic exploration of the specific mechanisms by which NEXN regulates ER stress in VSMCs under both physiological and pathological conditions. Future research will focus on elucidating the interplay between other signaling pathways, such as G protein signaling and ER stress, in NEXN-mediated VSMC phenotypic transition.

## Methods

### Sex as a biological variable.

For the mouse experiments, only males were used to avoid potential interference arising from the estrogenic activity of tamoxifen. The findings are expected to apply to both sexes.

A comprehensive overview of the antibody origins is outlined in Supplemental Table 1, with a detailed enumeration of the reagents employed in our study featured in Supplemental Table 2. The siRNA sequences designed to target NEXN in human samples are meticulously documented in Supplemental Table 3. Additionally, the primer sequences utilized for qRT-PCR analysis are displayed in Supplemental Table 4. This manuscript, along with its accompanying Supplemental Methods, provides a repository of all essential data, analytical methodologies, research materials, and relevant citations that underpin our findings.

### Cell culture and treatment.

Primary HASMCs were sourced from iCell Bioscience Inc. (catalog number: HUM-iCell-c010) and nurtured in a proprietary low-serum medium (PriMed-iCell-004) in strict adherence to the manufacturer’s guidelines. Our experimental work focused on HASMCs that were within the early passages of 2 to 8, unless explicitly noted otherwise. These cells were meticulously cultivated under controlled conditions in a humidified incubator at a temperature of 37°C, with an atmosphere of 5% CO_2_.

The SMC identity of HASMCs was confirmed through both morphological assessment and immunohistochemical staining using a monoclonal antibody directed against α–smooth muscle actin. Prior to experimentation, the HASMCs were subjected to serum deprivation to synchronize their cellular state. Subsequently, they were stimulated with either recombinant PDGF-BB at a concentration of 20 ng/mL (Recombinant Human PDGF-BB, AF-100-14B, PeproTech) or TGF-β at 10 ng/mL (Recombinant Human TGF-β1, 100-21, PeproTech), for a duration of 24 hours to induce cellular responses.

### Animal studies.

*Nexn^fl/fl^* mice were provided by Ju Chen from the Department of Medicine, University of California, San Diego, La Jolla, California, USA. *Myh11*-Cre/ER^T2^ mice and Rosa26-tdTomato mice were purchased from The Jackson Laboratory (Stock No. 019079, No. 007905). C57BL/6J mice were purchased from Beijing Vital River Laboratory Animal Technology Co., Ltd.

*Myh11*-Cre/ER^T2^ R26-tdTomato mice were generated by crossbreeding Rosa26-tdTomato mice with *Myh11*-Cre/ER^T2^ mice. *Nexn^fl/fl^* *Myh11*-Cre/ER^T2^ mice were generated by crossbreeding *Nexn^fl/fl^* mice with *Myh11*-Cre/ER^T2^ mice. As the *Myh11*-Cre transgene was randomly inserted in the Y chromosome in these animals, only male mice were used in this study. To generate the VSMC lineage-tracing (*Myh11*-Cre/ER^T2^ R26-tdTomato) mice and the *Nexn^ismKO^* mice, mice were treated with tamoxifen (Sigma-Aldrich, T5648) by intraperitoneal injection at 8 weeks old at a dose of 40 mg/kg/d for 5 days as previously described (12). All mice were genotyped by PCR amplification (Thermo Fisher Scientific, K1082) with tail DNA samples as previously described (11). The specific primers for genotyping are listed in Supplemental Table 5.

### Mouse carotid artery wire injury model.

Carotid artery wire injury was performed using a guide wire (Cook Medical, C-SF-15-15) to induce neointima formation as described previously (35). Briefly, 8- to 12-week-old mice were anesthetized using either 2.5% isoflurane or an intraperitoneal injection of sodium pentobarbital at a dosage of 50 mg/kg. The left common carotid arteries were then surgically exposed through a noninvasive dissection technique. Arterial blood flow was controlled, either by permanent ligation with 8-0 silk sutures or by temporary clamping. An arteriotomy was made in the external carotid artery using fine microscissors, and the blood was cleared with saline and cotton swabs. The guide wire was inserted 1 cm into the external carotid artery lumen and manipulated back and forth 10 times to ensure complete endothelial denudation. After wire withdrawal, the external carotid artery was ligated with an 8-0 silk suture. The right common carotid artery served as a control and was dissected without injury. The surgical wound was closed with 6-0 silk sutures, and mice were kept on a heating pad postsurgery. They were then allowed to recover before being returned to their cages. At 28 days postinjury, the mice were euthanized, and the carotid arteries were meticulously harvested and prepared for cryostat sectioning for subsequent analysis.

### Morphometric analysis and quantification of neointima formation.

Mice were humanely euthanized via an intraperitoneal injection of sodium pentobarbital at a dosage of 50 mg/kg, followed by perfusion with PBS to clear the vasculature of blood. The animals were then fixed with a 4% paraformaldehyde (PFA) solution. Both the left injured common carotid arteries and the right uninjured counterparts were meticulously harvested and subjected to overnight fixation in 4% PFA. The arterial samples were subsequently dehydrated in a graded sucrose solution, starting with 10% and 20% sucrose for 30 minutes each, followed by an overnight incubation in a 30% sucrose solution. Afterward, the tissues were embedded in OCT Tissue Tek. The OCT-embedded arterial tissues were sectioned into 7 μm frozen sections using a Leica CM3050S Microtome Cryostat. For histological analysis, these sections were stained with VVG (Solarbio, G1597) and H&E (Solarbio, G1120) staining kits, adhering to the manufacturer’s protocols. The stained sections were then imaged using a 3DHISTECH Slide Scanner to capture high-resolution images. Neointima formation, the neointima area, the media area, and the neointima/media ratio were measured using Fiji software, providing a detailed assessment of the arterial injury response.

### RNA extraction and library construction.

RNA was extracted and purified using TRIzol Reagent (Invitrogen, 15596026), and its quantity and purity were measured with a NanoDrop ND-1000. RNA integrity was ensured with an RNA integrity number score above 7.0, as assessed by Bioanalyzer 2100 (Agilent) and agarose gel electrophoresis. Poly(A) RNA was selected from total RNA using Dynabeads Oligo (dT) 25-61005 (Thermo Fisher Scientific) and fragmented. The fragments were reverse-transcribed into cDNA by SuperScript II Reverse Transcriptase (Invitrogen, catalog 1896649) and converted into U-labeled second-stranded DNAs. These were then prepared for adapter ligation by adding an A-base to the ends.

Indexed adapters were ligated to the A-tailed DNA, and the products were size-selected with AMPureXP beads. After UDG enzyme (New England BioLabs, catalog m0280) treatment, the products were amplified by PCR. The final cDNA library had an average insert size of 300 ± 50 bp. Sequencing was performed using a 2 × 150 bp paired-end approach on an Illumina NovaSeq 6000, following the manufacturer’s protocol.

### Bioinformatics analysis of RNA-Seq.

Fastp software (https://github.com/OpenGene/fastp; commit ID eb461d5) was used to filter out reads with adapter contamination, low quality, and undetermined bases and to verify sequence quality. HISAT2 (https://daehwankimlab.github.io/hisat2/) was used for read mapping to the *Homo sapiens* GRCh38 reference genome. StringTie (https://ccb.jhu.edu/software/stringtie) assembled the mapped reads and merged transcriptomes across samples to reconstruct a comprehensive transcriptome, which was further refined using gffcompare (https://github.com/gpertea/gffcompare/; commit ID f7b04b6). Expression levels were estimated with StringTie, calculating fold per million reads values for mRNAs. Differential expression analysis was conducted with edgeR, identifying mRNAs with a |log2*​*FC|>1 (FC > 2 or < 0.5) and a *P* < 0.05 from parametric *F* tests (Bioconductor; https://bioconductor.org/packages/release/bioc/html/edgeR.html).

All bioinformatic analyses, including GO, KEGG, and GSEA, were performed using OmicStudio tools (https://www.omicstudio.cn/tool) and R version 4.1.3 (2022-03-10). For GO and KEGG enrichment analysis, DEGs were mapped to GO terms in the GO database (http://www.geneontology.org/) and KEGG pathways, with significantly enriched terms (*P* < 0.05) identified using a hypergeometric test and visualized using the ggplot2 package (v3.3.3). GSEA was conducted using GSEA software (v4.1.0) and the Molecular Signatures Database, with genes ranked by their signal-to-noise ratios using the Signal2Noise metric in GSEA software. Terms with a |normalized enrichment score| > 1, nominal *P* < 0.05, and FDR *q* < 0.25 were considered significantly enriched and visualized using the ggplot2 package (v3.4.0).

### TEM.

For TEM analysis, HASMCs were collected and fixed with TEM fixative (Servicebio, G1102) for 2–4 hours at 4°C. Postfixation involved treatment with 1% OsO_4_ (Ted Pella, Inc. 18456) in 0.1 M PBS (pH 7.4) for 2 hours at room temperature. The cells were then centrifuged at 500*g* to pellet, pre-embedded in 1% agarose, and dehydrated through a graded ethanol series before being embedded in resin. The resin-embedded samples were polymerized at 65°C for over 48 hours. Ultrathin sections of 60–80 nm were cut on an ultramicrotome (Leica UC7), collected on 150 meshes cuprum grids with formvar film, and stained with 2% uranyl acetate and 2.6% lead citrate. After air-drying overnight, the ultrastructure was observed under a TEM device (HITACHI HT7700).

### Statistics.

Data were analyzed with GraphPad Prism 8.0 software. Data are presented as mean ± SEM, where each data point corresponds to 1 independent experiment or 1 individual animal. Data normality was assessed using a Shapiro-Wilk test. Statistical significance between 2 groups was analyzed using unpaired, 2-tailed Student’s *t* tests. Three or more groups were compared using 1-way ANOVA, followed by Dunnett’s multiple comparisons post hoc test with 95% CI. A value of *P* less than 0.05 was considered statistically significant.

### Study approval.

All animal experiments were conducted in compliance with the Guide for the Care and Use of Laboratory Animals issued by the Ministry of Science and Technology of China, approved by the Animal Ethics Committee of Zhujiang Hospital, Southern Medical University (LAEC-2022-250). All human protocols involved in this study adhered to the principles outlined in the Declaration of Helsinki and received approval from the Medical Ethics Committee of Zhujiang Hospital, Southern Medical University (No.2023-KY-209). Informed consent was obtained from all participants, and their personal information was anonymized to ensure confidentiality.

### Data availability.

The scRNA-Seq data used in this study were from a public dataset that had been deposited in the NCBI Gene Expression Omnibus (GEO) and are accessible through the GEO Series accession number GSE182232. DEGs for TGF-β–treated and PDGF-BB–treated VSMCs were obtained from a published dataset (18) (Jia et al.; Supplemental Tables 1 and 2) and reanalyzed as shown in Figure 1B. The code required to replicate the scRNA-seq analysis of dataset GSE182232 can be accessed via the GitHub repository at https://github.com/NeurosurgeonWen/scRNA-seq-analysis-for-PMID40440261 (commit ID 4bc4b2aa2afd85076828f134b3a00cae3227efda). The RNA-Seq data generated in this study have been deposited in GEO (GSE297149). Values for all data points in graphs are reported in the Supporting Data Values file.

## Author contributions

HY, CL, and ZL developed the concept; designed the study; and wrote the manuscript. ZW and WG analyzed the data. HY, ZL, and ZW drafted the manuscript. HY, ZL, CW, and ZC performed experiments and acquired and processed data. X Fan, CW, and RZ provided clinical samples. X Feng, ZH, RZ, and X Fan provided technical assistance and reagents. The order of co–first authors was determined by the volume of work each contributed to the study.

## Supplementary Material

Supplemental data

Unedited blot and gel images

Supplemental table 6

Supporting data values

## Version 1 05/29/2025

In-Press Preview

## Version 2 07/08/2025

Electronic publication
