# Supplementary material for: NEXN regulates vascular smooth muscle cell phenotypic switching and neointimal hyperplasia
Source: JCI Insight. 2025 May 29;10(13):e190089. doi: 10.1172/jci.insight.190089 (PMC12288906; doi:10.1172/jci.insight.190089)
Supplement: Supplemental data [file jciinsight-10-190089-s009.pdf]

Supplemental Materials

NEXN regulates vascular smooth muscle cells phenotypic switching and neointimal hyperplasia

Authors:

Zexuan Lin[1,2*]; Chaojie Wang[3*]; Zhuohua Wen[4*]; Zhaohui Cai[5]; Wenjie Guo[1,2]; Xin Feng[4]; Zengyan Huang[6]; Rongjun Zou[3]; Xiaoping Fan[3#]; Canzhao Liu[1, 2#], Hanyan Yang[1, 2#]

CONTENT

Expanded Materials and Methods

Supplemental Tables S1-S6

Supplemental Figures S1-S4

Supplemental References 1-4

Expanded Materials and Methods

scRNA-seq data processing and contractile score calculation

Single-cell RNA sequencing (scRNA-seq) data were obtained from the GEO database (GSE182232), comprising cells from sham-operated mouse femoral arteries and those subjected to wire-induced injury for 2 or 4 weeks.(1) Integration, quality control, interpretation, and clustering of the scRNA-seq data were performed using Seurat (5.1.0). Cells with fewer than 200 or more than 6,000 detected genes, or with more than 10% mitochondrial counts, were filtered out. Markers used to identify VSMCs clusters were based on gene signatures provided by the original study.(1) For cells within the VSMCs clusters, contractile gene signatures-including *ACTA2*, *MYH11*, and *TAGLN*- were used to calculate contractile scores using the AddModuleScore function in Seurat.

Screening candidate genes correlated with contraction score and associated with VSMCs phenotypic switching

For each gene expressed in at least 20% of VSMCs, we fitted a linear mixed-effects model where the output variable was the contraction score and the input was the normalized and scaled gene expression counts. The beta coefficient for the fixed effect of each input gene measured gene-specific changes in the contraction score, while adjusting for housekeeping genes (ACTB and B2M) and incorporating mouse samples as a random effect term for robust estimation. We also performed differential expression analysis to investigate the expression changes of these genes by comparing VSMCs from injured femoral arteries to those from sham-operated controls. Genes with adjusted $P < 0.05$ and meeting the thresholds of beta coefficient > 0.20 and log2[fold change (FC)] < -1, or beta coefficient < -0.20 and log2[FC] > 1, were considered significantly positively or negatively correlated with contraction scores, respectively.

To confirm the expression changes associated with VSMCs phenotypic switching, we

compared our significantly positively correlated genes with the upregulated genes in TGF-β-treated rat VSMCs and the significantly negatively correlated genes with the downregulated genes in PDGF-BB-treated rat VSMCs.(2) Genes with $|log2[FC]| > 1$ and adjusted $P < 0.05$ were considered significantly differentially expressed. Linear mixed-effects models were performed using lme4 (1.1.35.5), contraction scores were calculated using the AddModuleScore function in Seurat (5.1.0), and differential gene expression analysis was conducted using DESeq2 (1.44.0).

Analysis of NEXN expression along the pseudotime trajectory of VSMCs phenotypic switching

We reclustered the analyzed VSMCs from the integrated samples—including sham-operated controls and 2- or 4-week after wire-induced injury of the mouse femoral arteries — into three groups: contractile VSMCs, de-differentiated VSMCs, and synthetic/proliferative VSMCs. These groups were distinguished by their enrichment in contractile and synthetic VSMCs markers. The extracted VSMCs were converted to a SingleCellExperiment object using the as.SingleCellExperiment function in monocle3 (1.3.7). Trajectory inference was performed using Slingshot (2.12.0) with default settings. After extracting pseudotime values for the VSMCs, NEXN expression along the pseudotime trajectory was visualized using ggplot2 (3.5.1).

siRNA transfection

Small interfering RNA (siRNA) oligonucleotides targeting human NEXN along with a non-specific scrambled siRNA control were expertly crafted and synthesized by RiboBio. HASMCs were transfected with siRNAs (25 nM) in vitro using Lipofectamine 3000 Transfection Reagent (L3000015, Invitrogen) and Opti-MEM™ I Reduced Serum Medium (31985070, Gibco) according to the manufacturer's protocol. A comprehensive list of the siRNA sequences can be found in Supplemental Table S3.

The VSMCs were transfected with si-NEXN and negative control (NC) siRNA for 24 hours, followed by stimulation with PDGF-BB for 24 hours in medium containing 2% FBS.

Immunofluorescence analysis

Human arterial samples and mouse carotid arteries were rinsed with PBS and fixed overnight in a 4% PFA solution to preserve tissue integrity. The arteries were then dehydrated in 10% and 20% sucrose solutions for 30 minutes each, followed by immersion in a 30% sucrose solution overnight. Afterwards, the dehydrated tissues were embedded in OCT tissue tek and cut into 7-μm thick cryo-sections. VSMCs were fixed with 4% PFA for 15 minutes to maintain cellular structure. Both the cryo-sections from human arteries, mouse carotid arteries, and HASMCs were washed with PBS (3 × 5 minutes) and incubated in a blocking solution containing PBS with 5% BSA, 5% donkey serum, and 0.1% Triton for 1 hour. Primary antibodies were applied overnight, followed by a 1-hour incubation with secondary antibodies, as previously described.(3) Nuclei were stained with DAPI (H-1200-10, Vector Laboratories). Primary and secondary antibodies are listed in Supplemental Table S1. Immunofluorescence images were acquired using a Leica DMi8 confocal microscope and processed using Fiji software and Adobe Illustrator 2021.

Protein isolation and western blot analysis

Proteins were extracted and subjected to immunoblotting as previously described.(4) Briefly, cells and tissues were lysed with RIPA buffer (P0013, Beyotime) supplemented with 1% protease inhibitor (539131, Merck Millipore) kept on ice. The lysates were then centrifuged at 12,000 rpm for 15 minutes at 4°C to remove debris and obtain a clear supernatant. Equal quantities of total protein were separated on 10% SDS-PAGE gels (Epizyme, PG212) and transferred onto a PVDF membrane (Millipore,

IPVH00010) for 1.5 hours at 4°C. The membranes were subsequently blocked in TBST buffer containing 5% skimmed milk powder for 1 hour and then incubated with primary antibodies at 4°C overnight, followed by incubation with the corresponding HRP-conjugated secondary antibodies (either anti-rabbit or anti-mouse) for 1 hour at room temperature. A list of the primary and secondary antibodies used can be found in Supplemental Table S1. The immunoreactive bands were visualized using an enhanced chemiluminescence (ECL) detection system (Millipore, WBKLS0100), and the resulting chemiluminescent signals were captured with an Alliance Q9 imaging system (Uvitec).

Quantitative real time PCR (qRT-PCR) analysis

Total RNA was isolated from HASMCs using either TRIzol Reagent (Invitrogen, 15596026) or the SevenFast Total RNA Extraction Kit for Cells (SEVEN, SM130-02). For reverse transcription, 500 ng of total RNA was converted into cDNA using the PrimeScript™ RT Master Mix (Takara, RR036A). Quantitative RT-PCR was conducted with TB Green fluorescence dye (Takara, RR820A), following the manufacturer's guidelines. Each reaction mixture for the real-time PCR contained 2 μL of the reverse transcription product, 12.5 μL of TB Green, and 0.4 μM of both forward and reverse primers, with primer sequences detailed in Supplemental Table S4. The PCR amplification was performed on a Fast 96-Well System (Applied Biosystems), consisting of 40 cycles with a denaturation step at 95 °C for 5 seconds, annealing at 60 °C for 30 seconds, preceded by an initial denaturation at 95 °C for 30 seconds. Each sample was analyzed in duplicate, and the RNA was prepared from three separate experiments to ensure reproducibility.

Cell cycle analysis

To overexpress NEXN, Ad- NEXN or VEC adenovirus was utilized to infect HASMCs

for 24 hours. To knock down NEXN, human si-NEXN or NC siRNA was transfected into HASMCs for 24 hours, followed by stimulation with PDGF-BB for additional 24 hours. Cells were collected and fixed with 70% ethanol, followed by staining with Cell Cycle and Apoptosis Analysis Kit (Beyotime, C1052) following the manufacturer's instructions. Flow cytometric analysis was performed using a flow cytometer (CytoFLEXS, Beckman Coulter), and the data were analyzed using ModFitLT5 Software.

5-ethynyl-2'-deoxyuridine (EdU) incorporation assay

Cell proliferation was assessed using the Click-iT™ EdU Cell Proliferation Kit with Alexa Fluor™ 594 dye (Invitrogen, C10339), following the manufacturer's guidelines. HASMCs were treated as described in the main text and then exposed to EdU (Invitrogen, A10044) for 2 hours prior to fixation and permeabilization. The cells were fixed with 4% PFA for 15 minutes and subsequently permeabilized with a solution containing 5% BSA, 5% donkey serum, and 0.1% Triton X-100 in PBS for 1 hour at room temperature. EdU staining was then performed according to the kit's instructions. Fluorescent images were captured using a Leica DMi8 confocal microscope, and image processing was conducted using Fiji software and Adobe Illustrator 2021.

Scratch wound healing assay

HASMCs were subjected to the treatments detailed in the main text. A scratch wound assay was performed by creating a scratch in the cell monolayer with a sterile pipette tip ranging from 10-20 μL, and initial photomicrographs were captured using a Leica microscope. Following stimulation with PDGF-BB, subsequent images were taken at 6, 12, 16, and 24 hours to track VSMCs migration across the wounded area. The percentage of wound closure was then calculated using Fiji software.

Supplemental Table S1. Antibodies used in this study.

Antibody	Applications	Company	Catalog No.
Anti-Smooth muscle myosin heavy chain 11	WB (1:1000), IF (1:100)	Abcam	ab82541
Anti-Calponin 1 antibody	WB (1:1000)	Abcam	ab46794
Anti-Vimentin antibody	IF (1:100)	Abcam	ab8978
Anti-Nexilin/F-actin-binding antibody	WB (1:1000), IF (1:100)	Abcam	ab233267
Anti-Hsp90 antibody	WB (1:1000)	Abcam	ab203126
Anti-KLF4 antibody	WB (1:1000)	Abcam	ab272860
α-Smooth Muscle Actin (1A4) Mouse mAb	IF (1:100)	CST	48938
Anti-mouse IgG, HRP-linked Antibody	WB (1:2000)	CST	7076
Anti-rabbit IgG, HRP-linked Antibody	WB (1:2000)	CST	7074
Alexa Fluor® 488-AffiniPure Donkey Anti-Rabbit IgG (H+L)	IF (1:200)	Jackson	711-545-152
Alexa Fluor® 488-AffiniPure Donkey Anti-Mouse IgG (H+L)	IF (1:200)	Jackson	715-545-150
Alexa Fluor® 594-AffiniPure Donkey Anti-Rabbit IgG (H+L)	IF (1:200)	Jackson	711-585-152

Alexa Fluor® 594-AffiniPure Donkey Anti-Mouse IgG (H+L)	IF (1:200)	Jackson	715-585-150
Alexa Fluor® 647-AffiniPure Donkey Anti-Rabbit IgG (H+L)	IF (1:200)	Jackson	711-605-152
Alexa Fluor® 647-AffiniPure Donkey Anti-Mouse IgG (H+L)	IF (1:200)	Jackson	715-605-150
Nexilin Polyclonal antibody	WB (1:1000), IF (1:100)	Proteintech	27737-1-AP
Beta Actin Monoclonal antibody	WB (1:2000)	Proteintech	66009-1-Ig
GAPDH Monoclonal antibody	WB (1:2000)	Proteintech	60004-1-Ig
transgelin/SM22 Polyclonal antibody	WB (1:1000)	Proteintech	10493-1-AP
CHOP; GADD153 Monoclonal antibody	WB (1:3000)	Proteintech	66741-1-Ig
ATF4 Polyclonal antibody	WB (1:1000)	Proteintech	10835-1-AP
KLF4 Polyclonal antibody	WB (1:1000)	Proteintech	11880-1-AP
β-tubulin（MG7）Mouse Monoclonal Antibody	WB (1:5000)	Beijing Ray Antibody	RM2003
Anti-Actin, alpha-Smooth Muscle antibody	WB (1:5000)	Sigma	A5228

Abbreviation: **WB**, western blotting; **IF**, immunofluorescence.

Supplemental Table S2. Reagents used in this study.

Reagent	Applications	Company	Catalog No.
Human primary aortic smooth muscle cells	cell	Bioscience Inc	HUM-iCell-c010
Primary smooth muscle cell low serum culture system	cell culture	Bioscience Inc	PriMed-iCell-004
Dulbecco's Modified Eagle Medium	cell culture	Biological Industries	06-1055-571ACS
Fetal bovine serum	cell culture	Gibco	10099141
Recombinant Human PDGF-BB	Growth Factors & Cytokines	PeproTech	AF-100-14B
Recombinant Human TGF-β1 (HEK293 derived)	Growth Factors & Cytokines	PeproTech	100-21
Lipofectamine 3000 Transfection Reagent	Transfection	Invitrogen	L3000015
Opti-MEM™ I Reduced Serum Medium	Transfection	Gibco	31985070
Antifade Mounting Medium with DAPI	IF	Vector Laboratories	H-1200-10
DAPI Staining Solution	IF	Beyotime	C1005
Cell lysis buffer for Western and IP	Protein isolation	Beyotime	P0013
Protease Inhibitor Cocktail Set I	Protein isolation	Merck Millipore	539131
Pierce™ BCA Protein Assay Kits	Protein Assays	Thermo Scientific	23227

Omni-Easy™One-Step PAGE Gel Fast Preparation Kit	Western blot analysis	Epizyme	PG212
PVDF Transfer Membrane	Western blot analysis	Millipore	IPVH00010
Immobilon Western Chemiluminescent HRP Substrate	Western blot analysis	Millipore	WBKLS0100
TRIzol Reagent	RNA Extraction	Invitrogen	15596026
SevenFast Total RNA Extraction Kit for Cells	RNA Extraction	SEVEN	SM13002
PrimeScript™ RT Master Mix	RNA reverse transcription	Takara	RR036A
TB Green® Premix Ex Taq™ II	qRT-PCR	Takara	RR820A
DreamTaq Green PCR Master Mix (2X)	PCR for genotyping	Thermo Scientific	K1082
Fixed Core Wire Guides	Carotid artery wire injury model	Cook Medical	C-SF-15-15
Collagen Fiber and Elastic Fiber Staining Kit	Morphometric Analysis	Solarbio	G1597
Hematoxylin-Eosin (HE) Stain Kit	Morphometric Analysis	Solarbio	G1120
Cell Cycle and Apoptosis Analysis Kit	Cell cycle analysis	Beyotime	C1052
EdU (5-ethynyl-2’-deoxyuridine)	Cell Proliferation	Invitrogen	A10044
Click-iT® EdU Imaging Kits	Cell Proliferation	Invitrogen	C10339

142

Supplemental Table S3. siRNA Sequences Targeting on human Genes

Product number	Gene	Target sequence
stB0014693B (work)	st-h-NEXN_001	GGAGATGATTCACTACTTA
stB0014693C (work)	st-h-NEXN_002	CCAGTCAGATTTACGGTTA

Supplemental Table S4. The primers used for qRT-PCR analysis

Gene	Homo sapiens
	Primers (5′-3′)
ACTA2	GCCAAGCACTGTCAGGAATC
	AAAACAGCCCTGGGAGCATC
TAGLN	GGAAACCCACCCTCTCAGTC
	TGCACTAGCCAAGTCATCCG
CNN1	GAGGCAAATAGCTGGGGTCC
	CCATCTTTGAGGCCGTCCAT
NEXN	ATGGCTCCACTGCTGAAGAT
	TTCTGGTTTGGGTTCTCCTG
GAPDH	GGAGCGAGATCCCTCCAAAAT
	GGCTGTTGTCATACTTCTCATGG

Supplemental Table S5. The primers used for mice genotyping

Gene	Primers (5′-3′)
Nexn$^{f/f}$	TCAAAGGGAAGGTCATTAAAATTC
	TGATGATGATGATGTTGCTAAGTG
Myh11-Cre/ER^{T2}	ATATAAGCCCAACTGCAGGAAA

	GTCAAAGTCAGTGCGTTCAAAG
Rosa26-tdTomato	Tomato-F CTGTTCCTGTACGGCATGG<br>Tomato-R GGCATTAAAGCAGCGTATCC<br>Rosa-WT-F AAGGGAGCTGCAGTGGAGTA<br>Rosa-WT-R CCGAAAATCTGTGGGAAGTC

Supplemental Figures

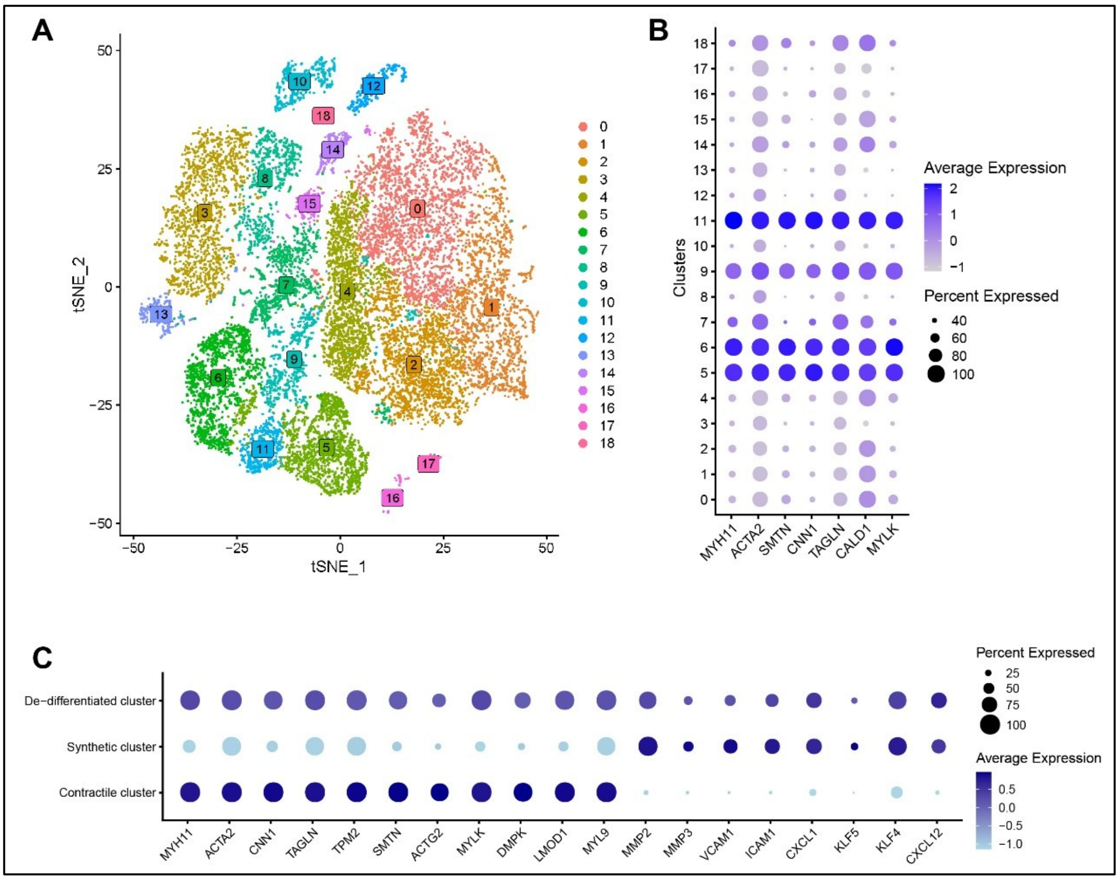

Figure S1. Identification of VSMCs clusters from single-cell transcriptomic data of mouse femoral arteries integrating sham-operated controls and 2- and 4- week post-injury samples (GSE182232).

A, t-SNE (t-distributed stochastic neighbor embedding) plot displaying the unsupervised clustering of 24,962 cells isolated from femoral arteries at all time points (sham-operated controls and 2 or 4 weeks after wire-induced injury). **B**, Expression of

VSMCs markers across 19 cell clusters. Clusters 5, 6, 9, and 11 were identified as VSMCs based on specific enrichment of classical contractile genes. **C**, Expression of contractile and synthetic VSMCs markers for the reclustered VSMCs extracted from **B**, showing three distinct groups: contractile VSMCs, de-differentiated VSMCs, and synthetic/proliferative VSMCs.

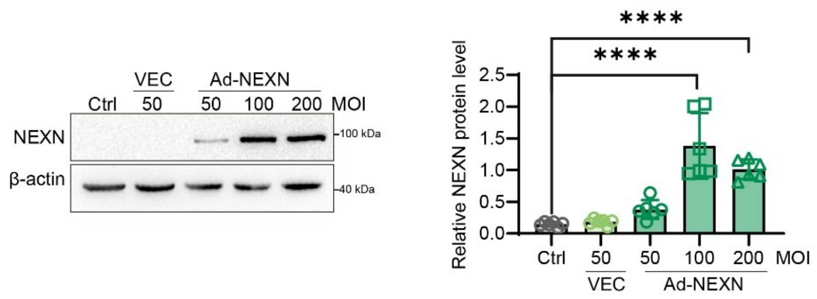

Figure S2. The overexpression efficiency of NEXN adenovirus was confirmed in vascular smooth muscle cells.

Representative Western blot (Left) and quantification (Right) of NEXN protein levels in HASMCs infected with VEC (50 moi) or NEXN adenovirus at 50, 100 and 200 moi respectively for 48 hours. β-actin was used as an internal control. n=6 for each group. Data are represented as mean ± SEM. Statistical analyses were performed using one-way analysis of variance. ****$P < 0.0001$ for indicated comparisons.

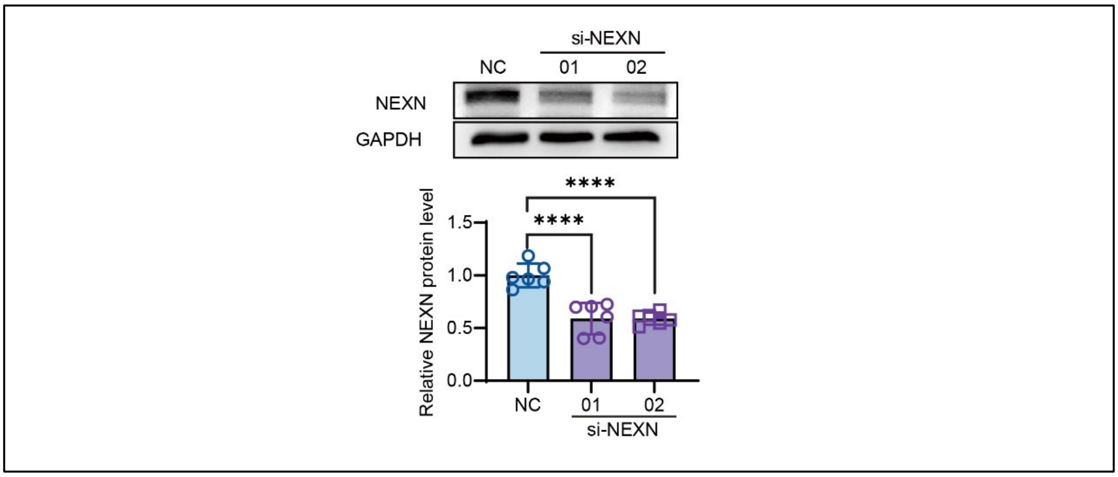

Figure S3. The knock-out efficacy of NEXN interference was validated in vascular smooth muscle cells.

Representative Western blot (Upper) and quantification (Lower) of NEXN protein levels in HASMCs transfected with scramble siRNA or NEXN single siRNAs (si-NEXN 01, 02) at 25 nM respectively for 48 hours. GAPDH was used as an internal control. n=6 for each group. Data are represented as mean ± SEM. Statistical analyses were performed using one-way analysis of variance. ****P < 0.0001 for indicated comparisons.

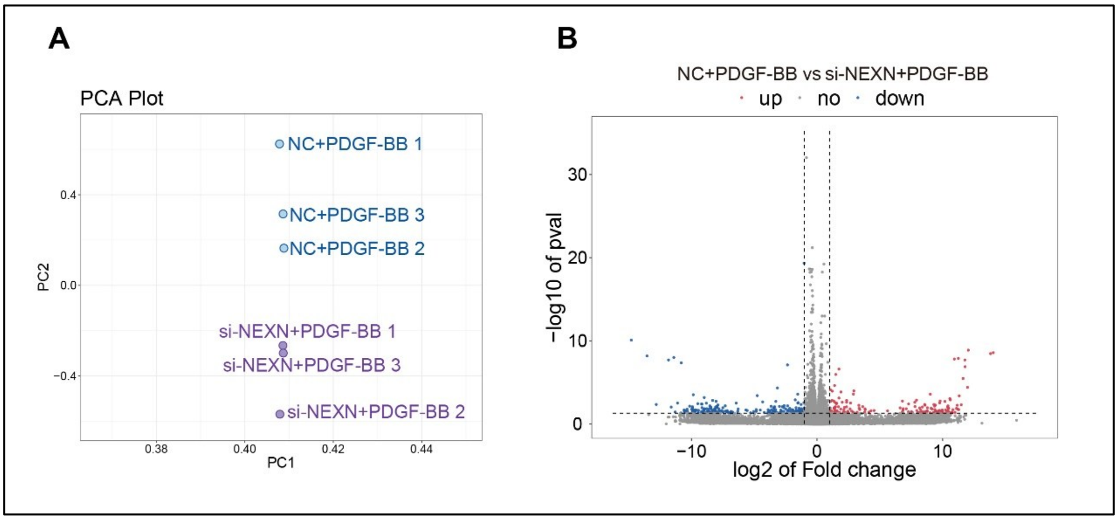

Figure S4. RNA-sequence analysis of gene expression from NC or si-NEXN pre-transfected HASMCs treated with PDGF-BB.

A, Principal component analysis (PCA) of RNA-seq data. NC+PDGF-BB and si-NEXN+PDGF-BB samples are labeled in blue and purple, respectively. **B,** Volcano plot of differentially expressed genes between NC+PDGF-BB and si-NEXN+PDGF-BB HASMCs, as revealed by RNA-seq.

References

1. Jiang L, et al. Nonbone marrow CD34(+) cells are crucial for endothelial repair of injured artery. *Circ Res*. 2021;129(8):e146-e165.
2. Jia Y, et al. PHB2 maintains the contractile phenotype of VSMCs by counteracting PKM2 splicing. *Circ Res*. 2022;131(10):807-824.
3. Liu C, et al. Nexilin is a new component of junctional membrane complexes required for cardiac T-Tubule formation. *Circulation*. 2019;140(1):55-66.
4. Yang HY, et al. Platelet CFTR inhibition enhances arterial thrombosis via increasing intracellular Cl(-) concentration and activation of SGK1 signaling pathway. *Acta Pharmacol Sin*. 2022;43(10):2596-2608.
