# Supplementary material for: NEXN regulates vascular smooth muscle cell phenotypic switching and neointimal hyperplasia
Source: JCI Insight. 2025 May 29;10(13):e190089. doi: 10.1172/jci.insight.190089 (PMC12288906; doi:10.1172/jci.insight.190089)

Full unedited gel for Figure 2A

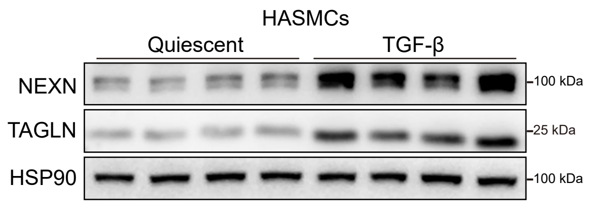

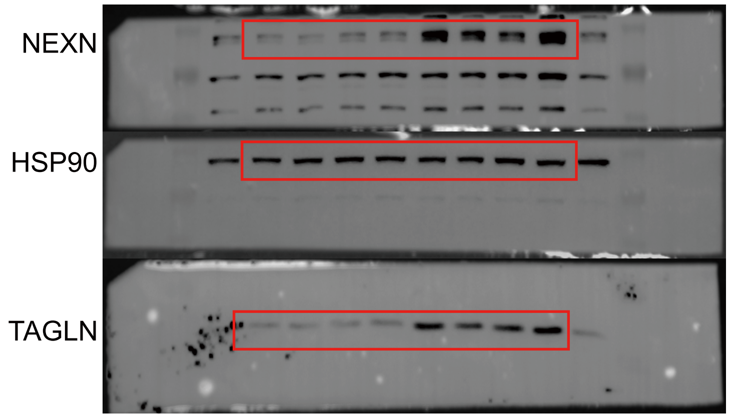

Full unedited gel for Figure 2B

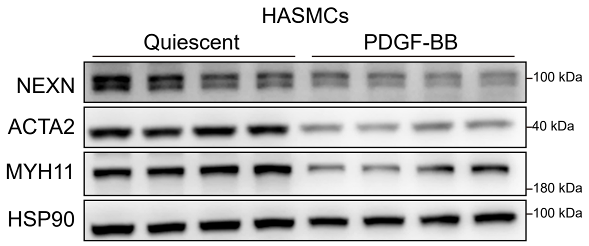

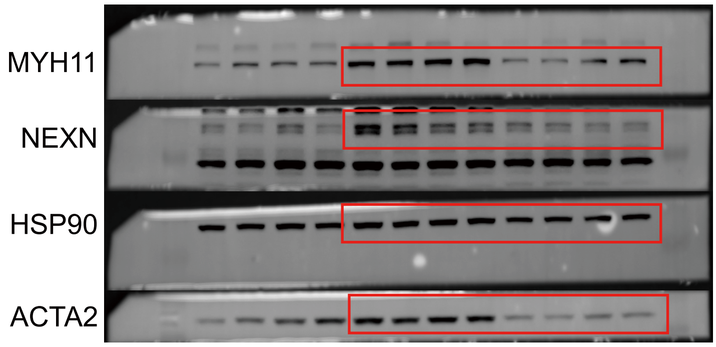

Full unedited gel for Figure 2C

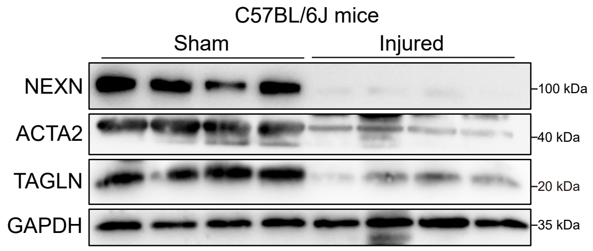

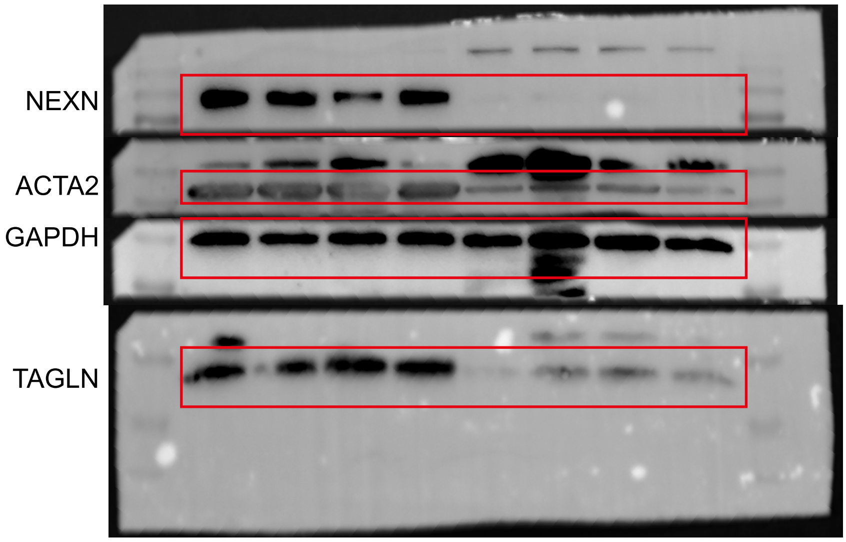

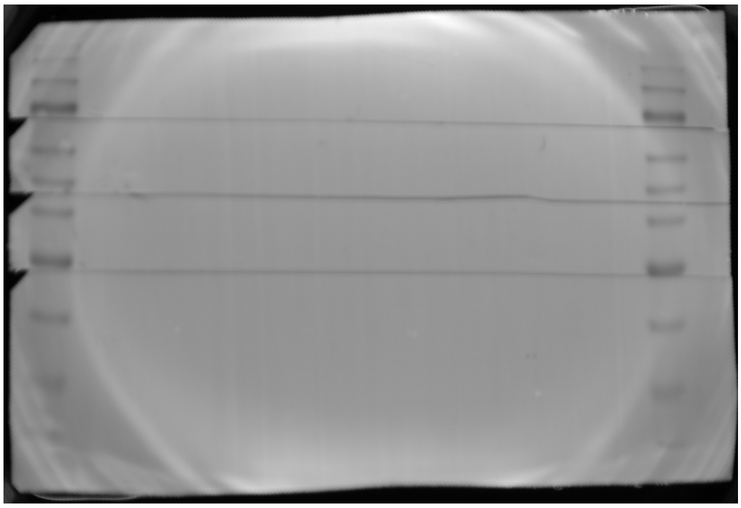

Full unedited gel for Figure 3A

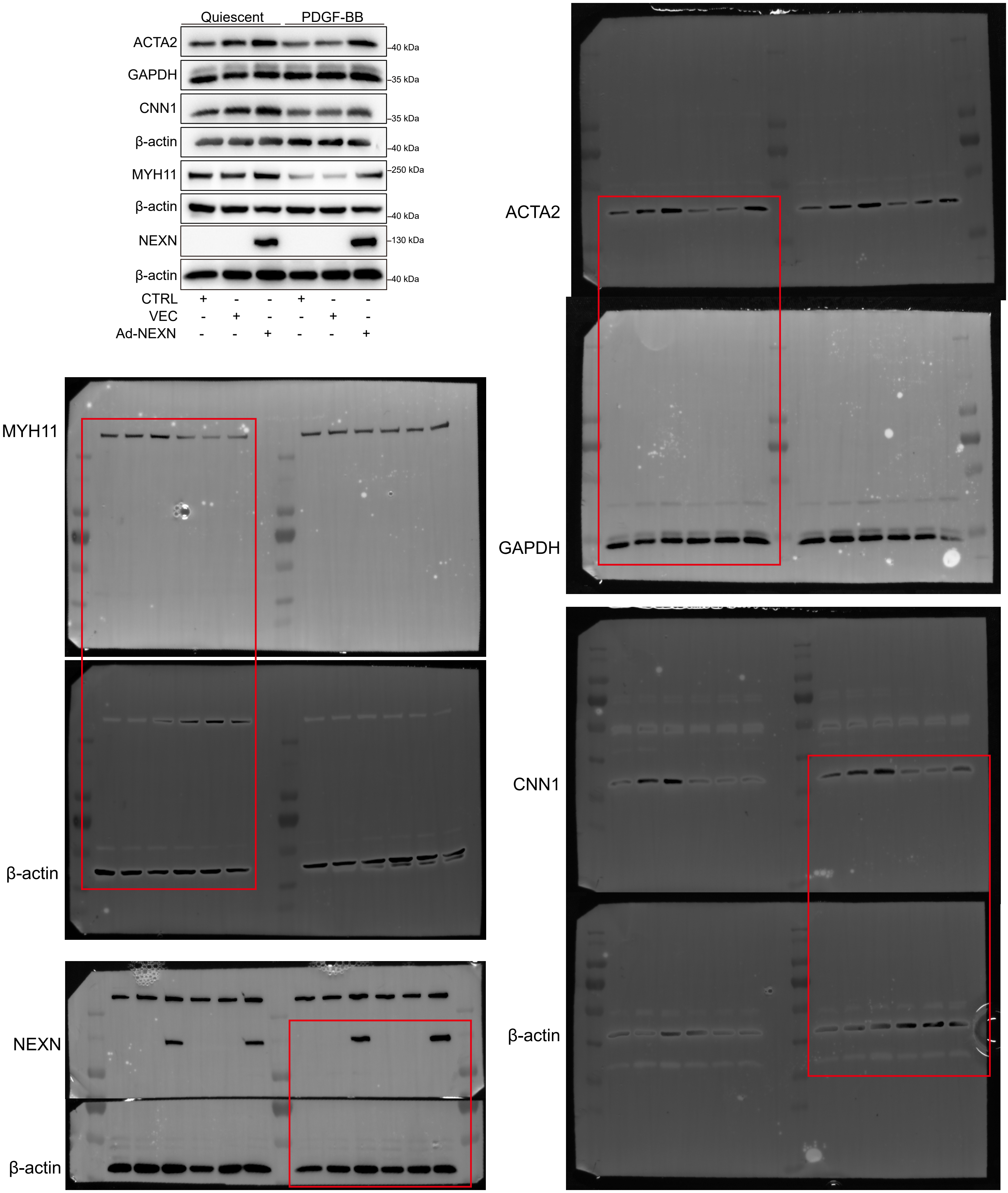

Full unedited gel for Figure 4A

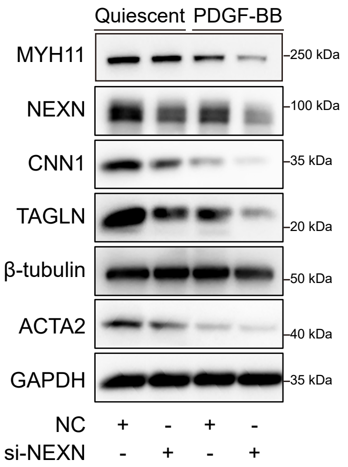

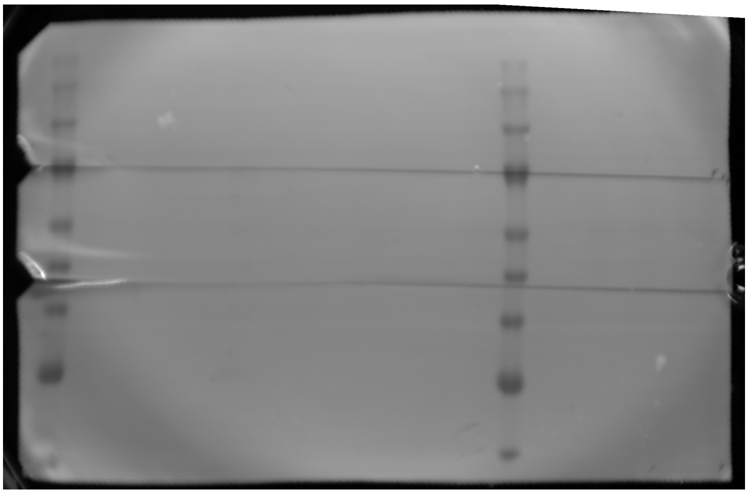

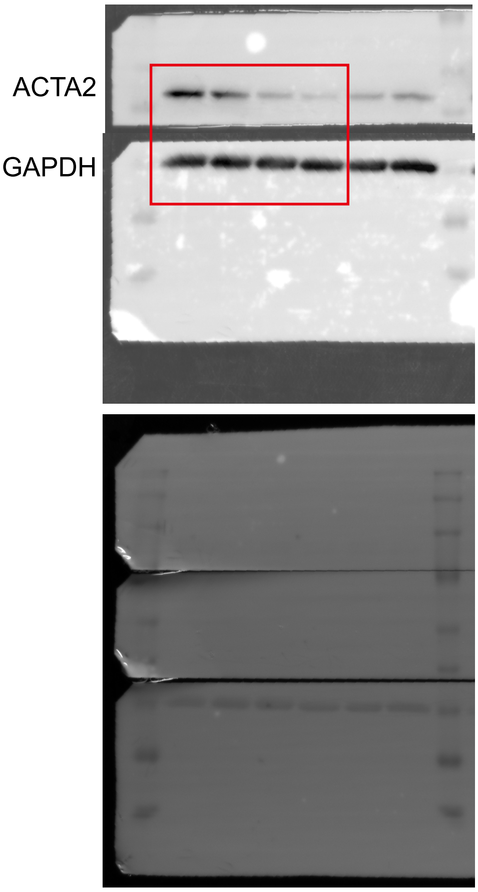

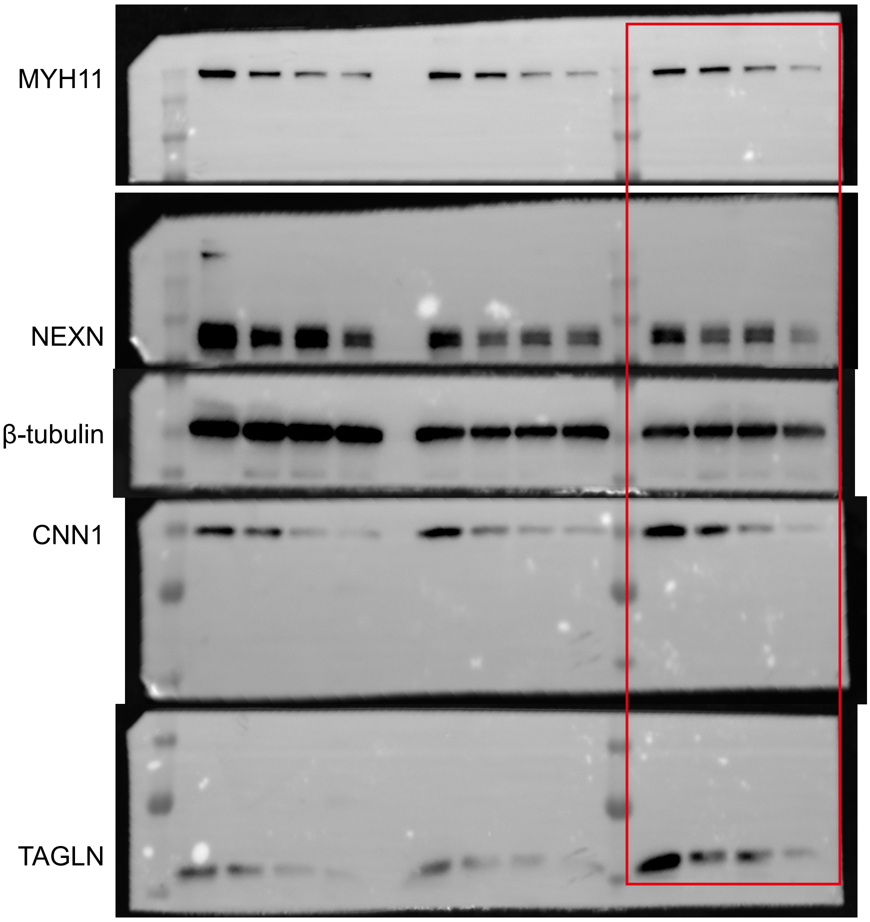

Full unedited gel for Figure 5B

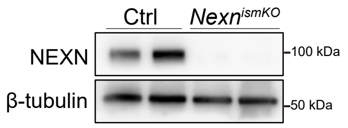

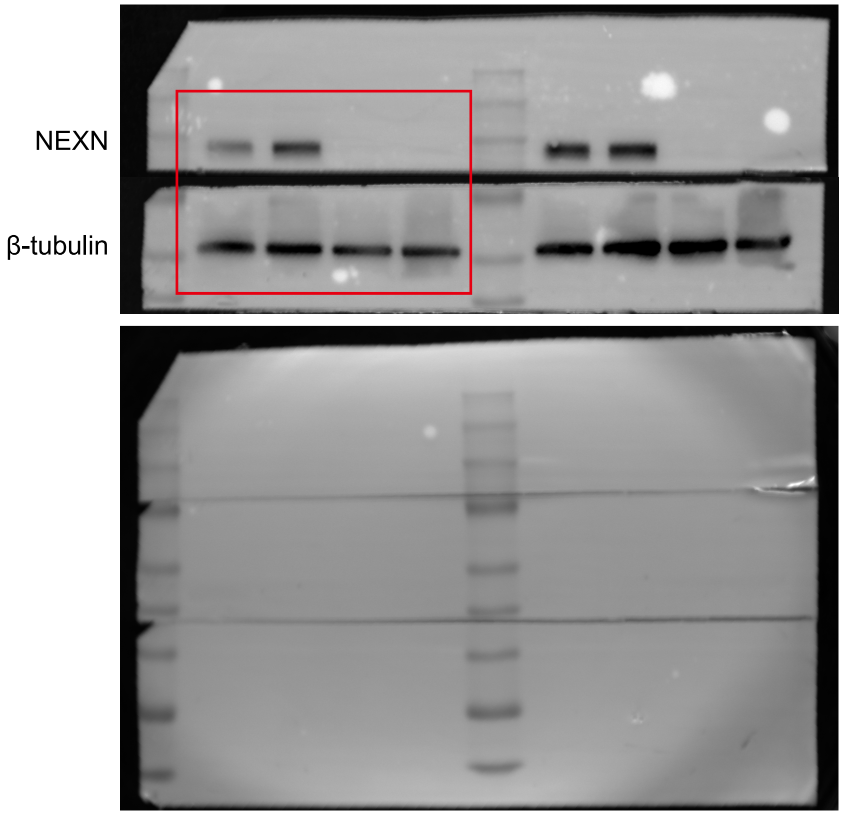

Full unedited gel for Figure 6E

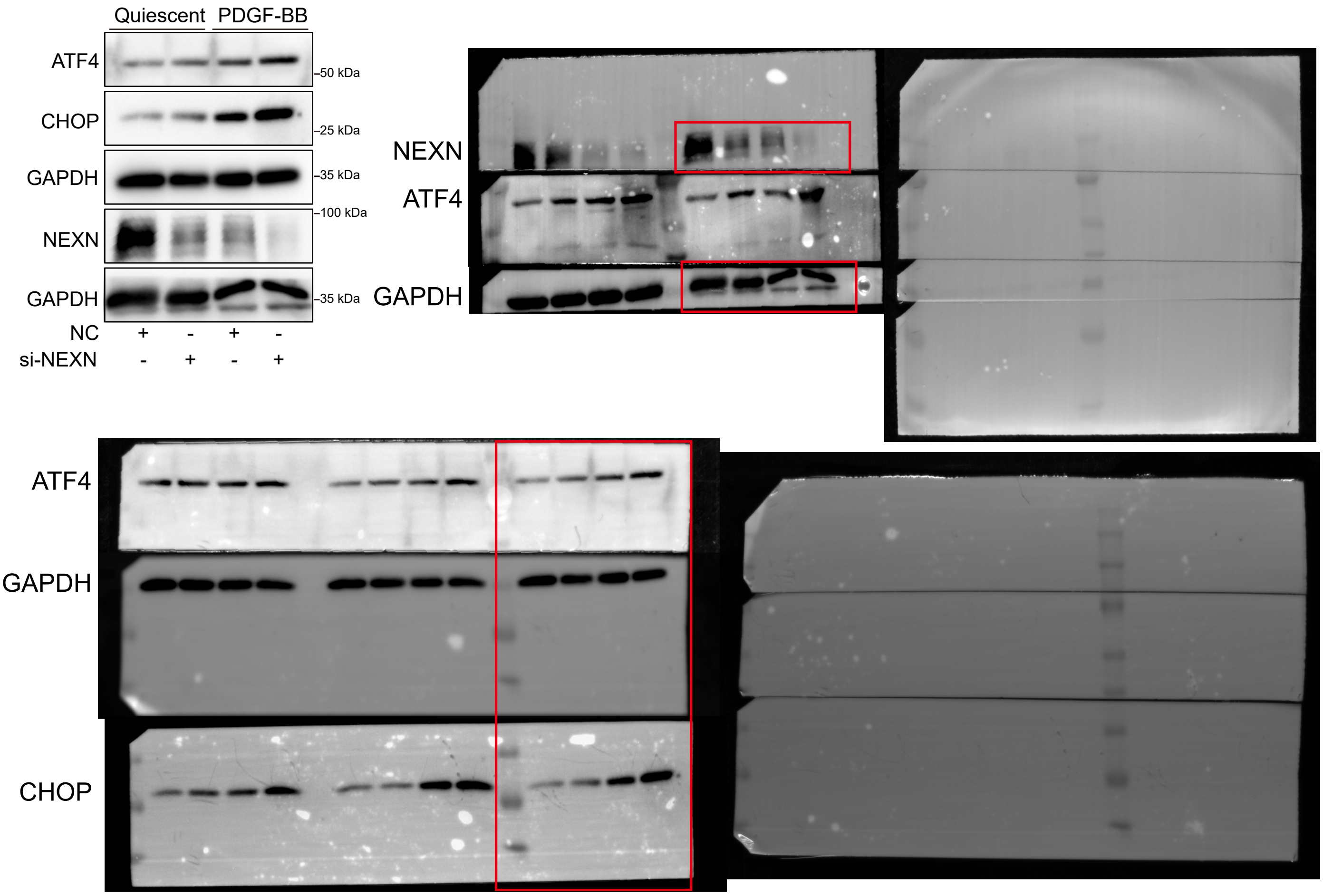

Full unedited gel for Figure 6F

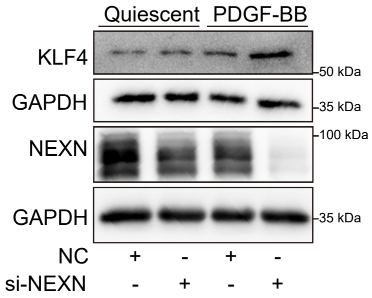

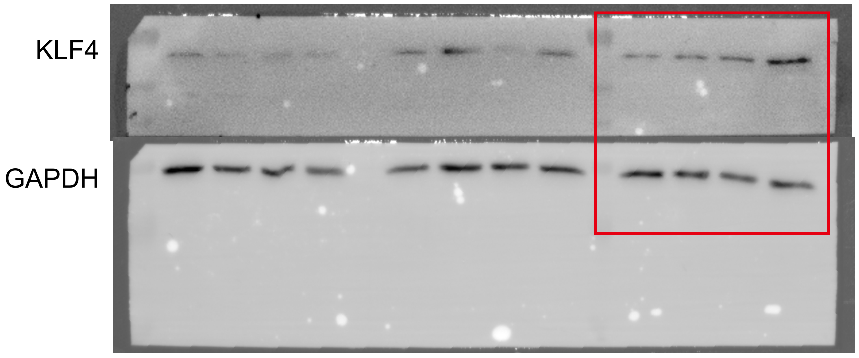

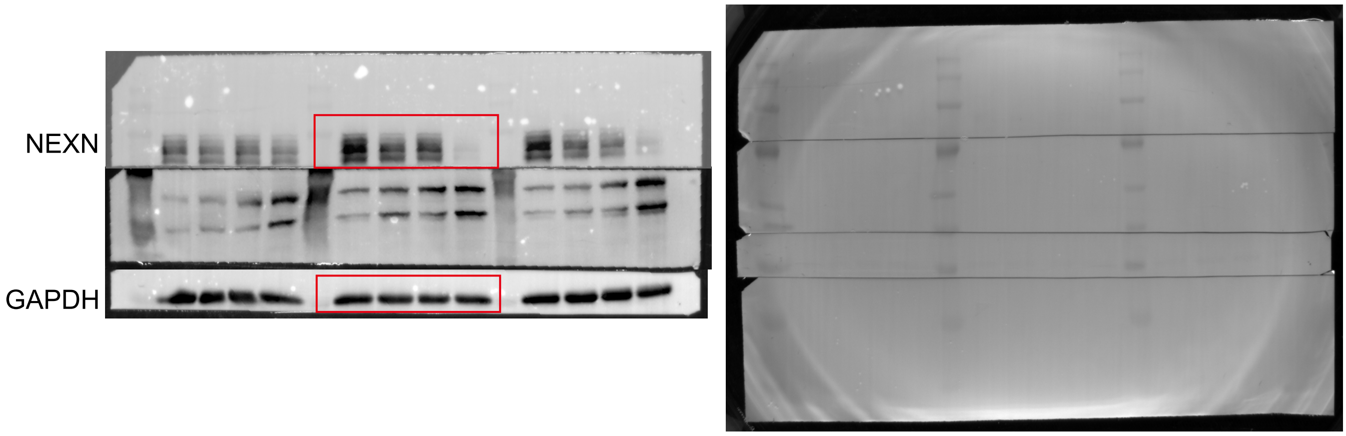

Full unedited gel for Figure 6G

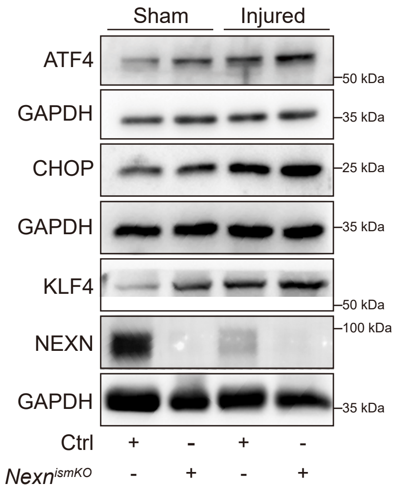

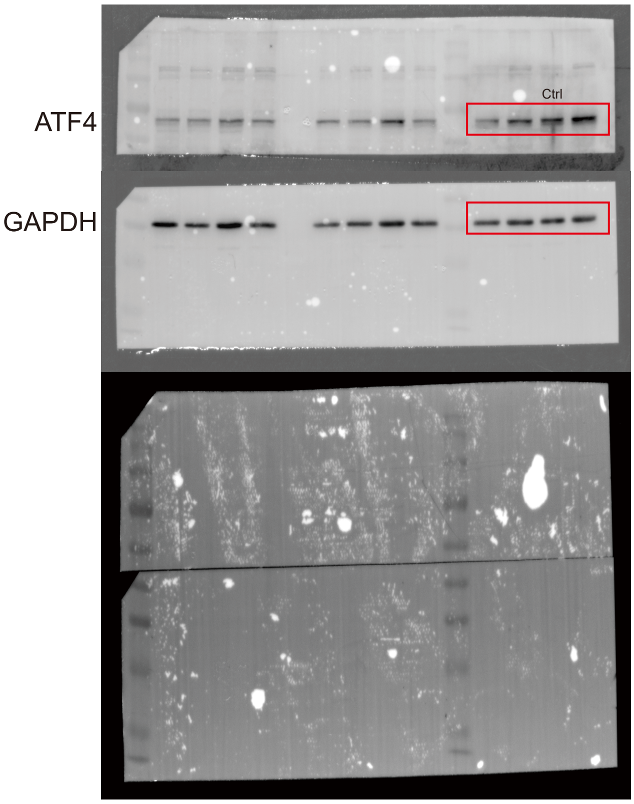

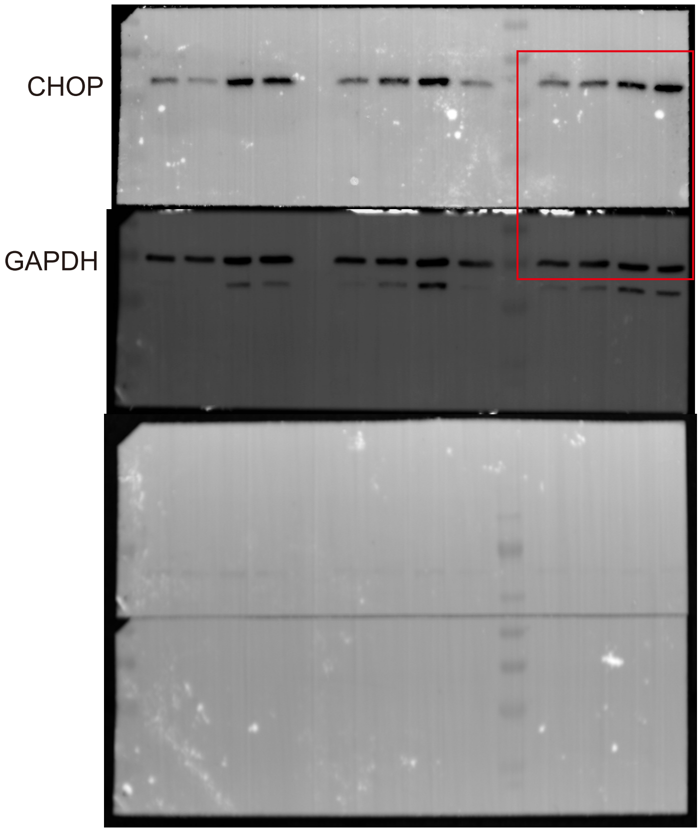

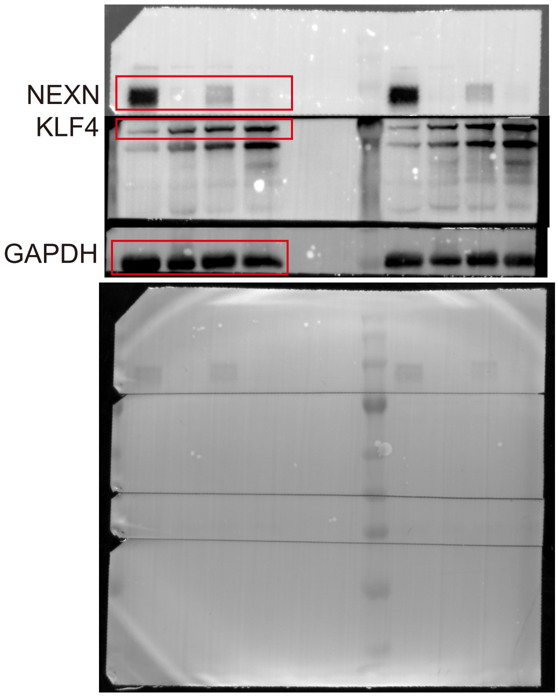

Full unedited gel for Figure 8A

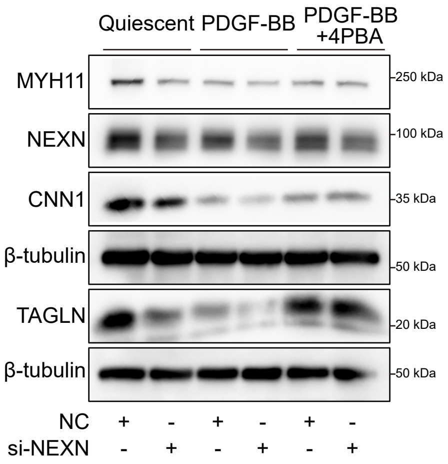

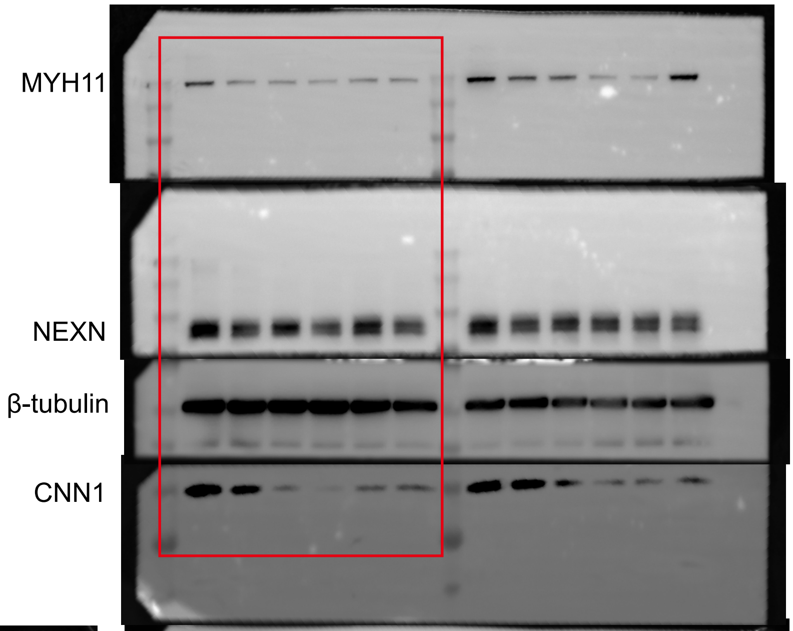

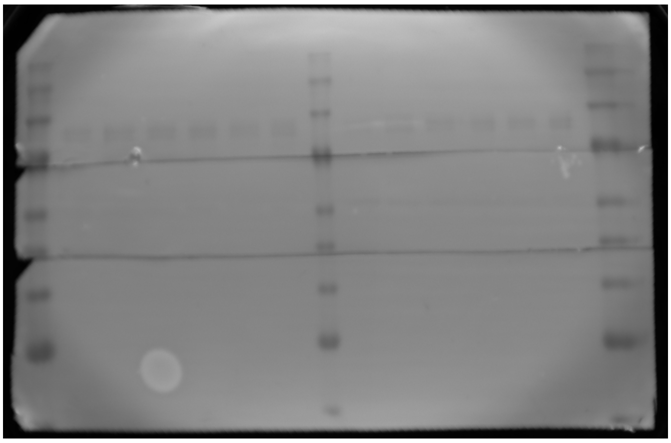

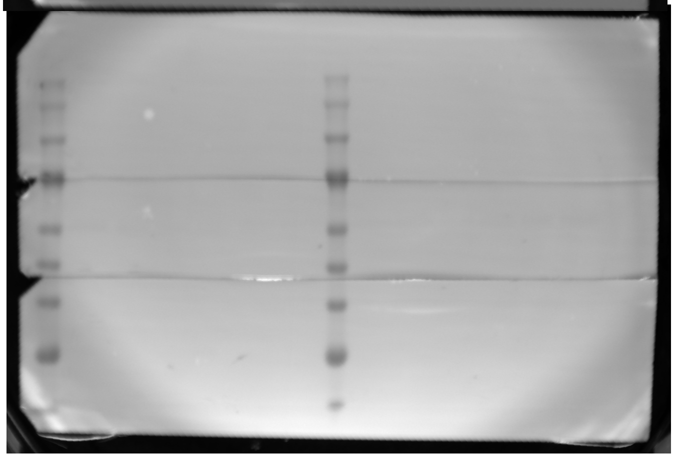

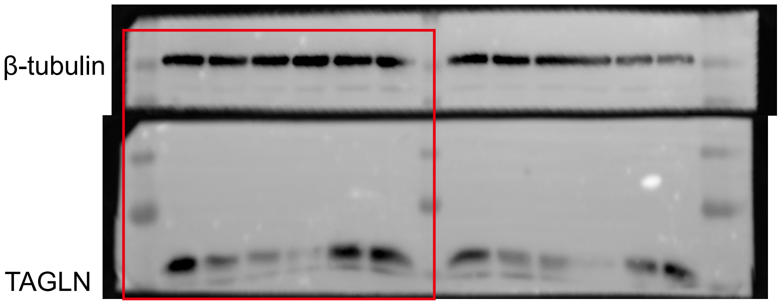

Full unedited gel for Figure 8G

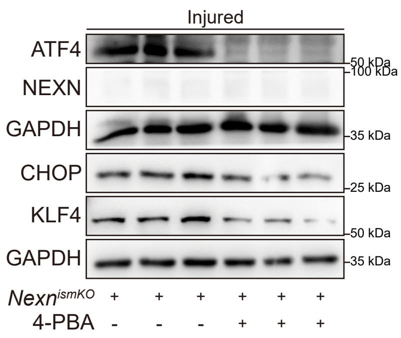

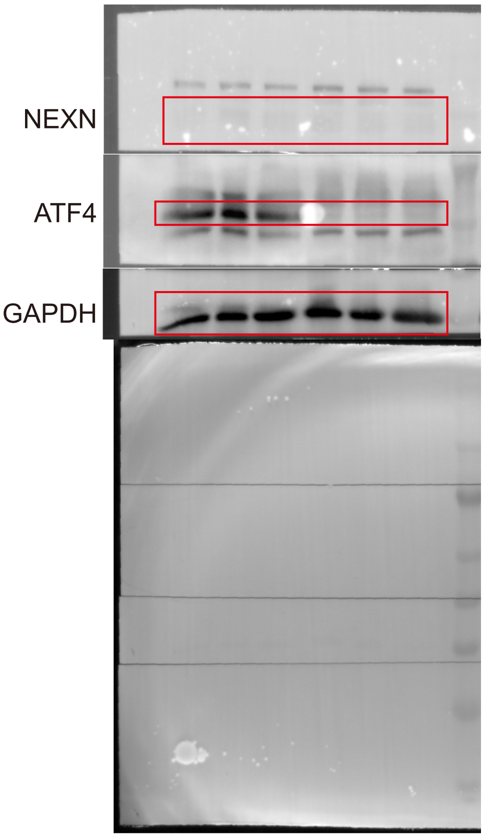

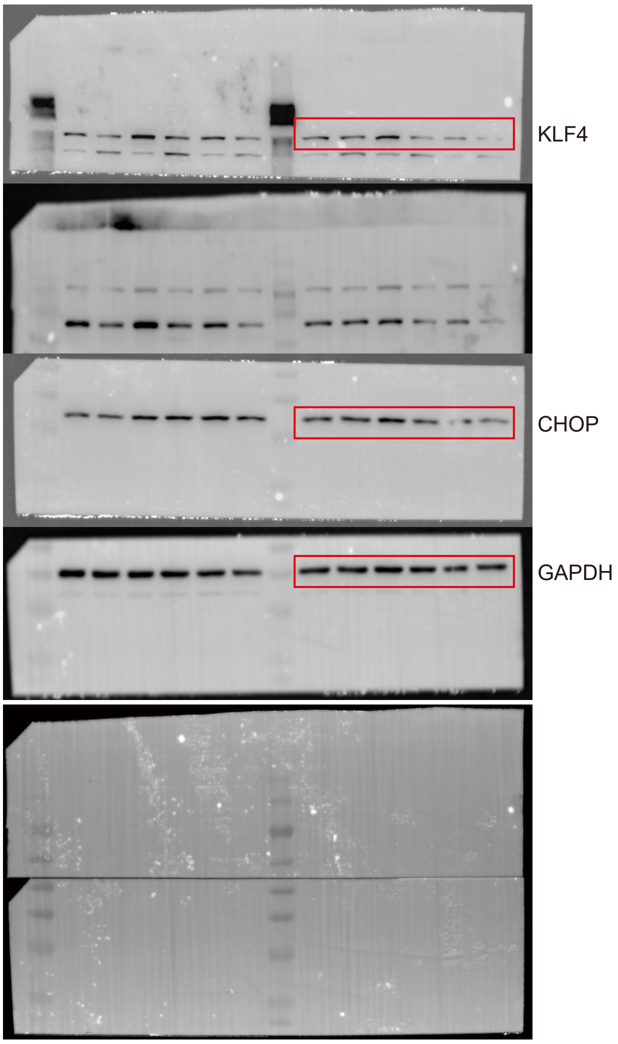

Full unedited gel for Figure S2

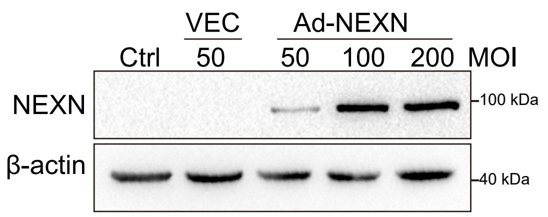

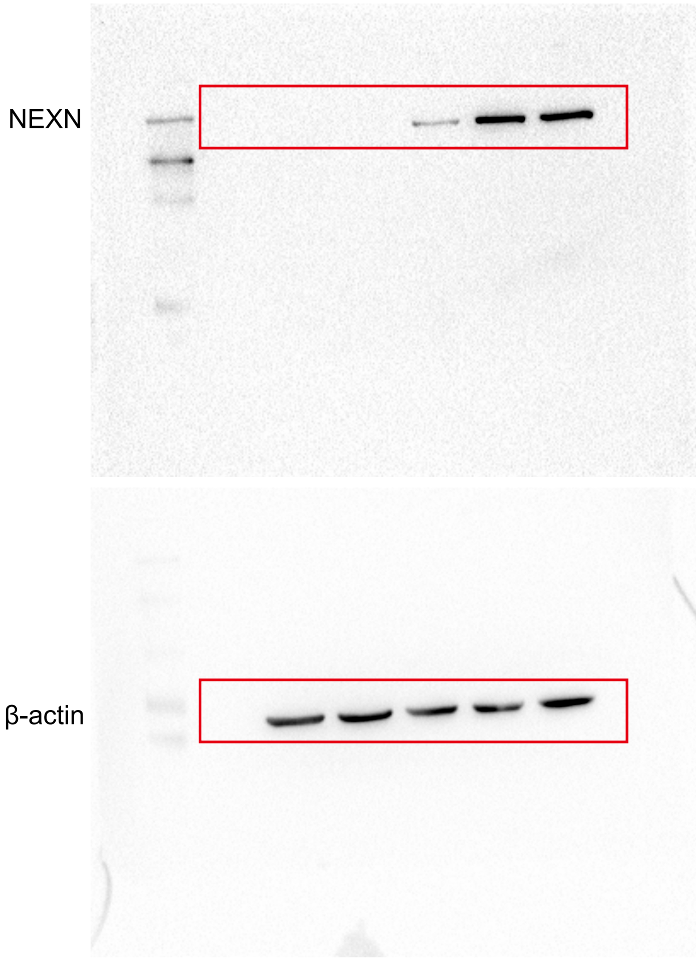

Full unedited gel for Figure S3

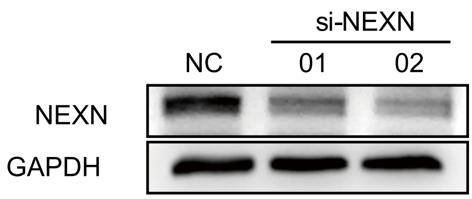

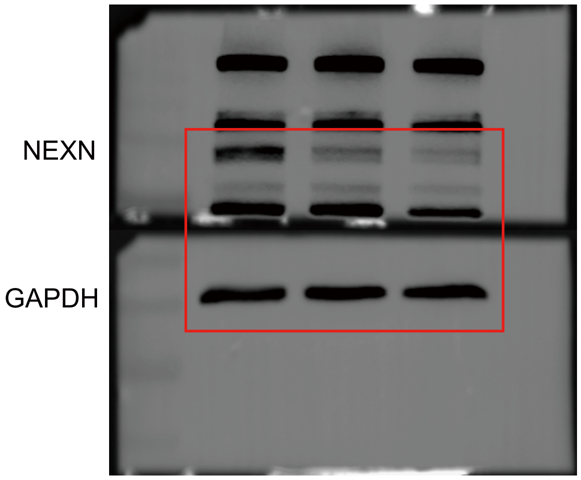

Supplement: Unedited blot and gel images [file jciinsight-10-190089-s010.pdf]
